# Simultaneous Screening and Quantification of 479 Pesticides in Green Tea by LC-QTOF-MS

**DOI:** 10.3390/foods12224177

**Published:** 2023-11-20

**Authors:** Xingqiang Wu, Yujie Xie, Kaixuan Tong, Qiaoying Chang, Xueyan Hu, Chunlin Fan, Hui Chen

**Affiliations:** Chinese Academy of Inspection and Quarantine, No. 11, Ronghua South Road, Beijing 100176, China; wuxq@caiq.org.cn (X.W.); xieyj@caiq.org.cn (Y.X.); tongkx@caiq.org.cn (K.T.); c81618@163.com (Q.C.); yuesehh@163.com (X.H.); caiqfcl@163.com (C.F.)

**Keywords:** green tea, pesticides, LC-QTOF-MS, SPE, high-throughput

## Abstract

A high-throughput screening and quantification method for 479 pesticides in green tea was established based on solid-phase extraction combined with liquid chromatography coupled with quadrupole time-of-flight mass spectrometry (LC-QTOF-MS). Pesticides were extracted from samples using an optimized SPE (TPT cartridges) procedure. LC-QTOF-MS in All Ions MS/MS scan mode acquired full MS data for quantification and product ion spectra for identification. LC-QTOF-MS quantification was achieved using matrix-matched standard calibration curves to achieve the optimal method accuracy. The method performance characteristics included the linearity, overall recovery, precision, and measurement uncertainty being evaluated. The validation results exhibited a good sensitivity with the LOQs of 5–55 µg/kg, which was satisfactory for their MRLs in China or the EU. The recoveries of more than 92.7% of the 479 pesticides in green tea were 70–120% at the three spiked levels with a precision of ≤20%. Finally, this method was employed to analyze 479 pesticides in 95 tea samples from markets in China. The test results of the tea samples showed that tolfenpyrad, buprofezin, and pyridaben were found with lower concentrations. The method has effectively improved the determination efficiency of pesticide residue screening by high-resolution mass spectrometry in green tea.

## 1. Introduction

Tea is one of the most popular drinks in the world, with green tea being one of the most consumed types of tea [1]. Tea has received much attention because of its particular nutrients (such as catechins, tea polyphenols, theanine, flavonoids, and essential minerals), which make it have anti-oxidation, anti-inflammation, and anti-cancer characteristics [2,3]. Global tea production has reached 6.497 million tons, while Asia accounted for 87.0% of this production, with China leading the way among many countries (2.792 million tons) [4]. With local characteristics, tea has become an important economic crop for poverty alleviation in China. As a perennial woody plant, tea is vulnerable to pests and weeds due to the high temperature and humidity of the cultivation environment. It has been reported that more than 800 species of pests and pathogens were found in tea plantations in China, which seriously affected the yield and quality of tea [5]. Therefore, the use of pesticides in the cultivation of tea trees is expected. With the growth of tea consumption, consumers are easily exposed to residual pesticides, resulting in potential health threats to consumers [6].

Pesticide residues in tea have an impact on product safety and trade circulation. Some countries and regional organizations, such as China, Japan, the European Union (EU), and the Food Codex Committee (CAC), have set stringent maximum residue limits (MRLs) for pesticides in tea. The EU made MRL value requirements for 516 possible pesticide residues in tea [7]. Japan has set MRLs for 230 pesticides in tea, while the pesticides that are not regulated must not exceed the uniform limit of 0.01 mg/kg limit value requirements [8]. China has set MRLs for 70 pesticides in tea with MRLs between 0.01 and 50 mg/kg [9]. It is necessary to meet the real need for high-throughput and high-sensitivity detection methods to ensure the safety of tea consumption and to reduce the economic losses caused by excessive pesticide residues in international trade.

Determining trace pesticide residues and high-throughput screening for numerous pesticides in complex food commodities remain challenges for analytical chemists. Improved multi-class or multi-residue methodologies with high sensitivity and expanded scopes, which include as many pesticides and commodities as possible in a single method, are required to check compliance or study the risk assessment of consumer pesticide exposure [10]. Indeed, sample pretreatment is a crucial aspect of pesticide residue determination in complex matrices. So far, many sample preparation methods for the analysis of pesticides in tea have been developed, including ultrasonic extraction (UE) [11], liquid–liquid extraction (LLE) [12], headspace solid-phase microextraction (HS-SPME) [13], dispersive liquid–liquid microextraction (DLLME) [14], accelerated solvent extraction (ASE) [15], matrix solid dispersion (MSPD) [16], dispersive solid-phase extraction (d-SPE) [17,18], QuEChERS [19,20], and solid-phase extraction (SPE) [21,22,23]. The reported methods for sample preparation in tea for multi-residues usually adopt QuEChERS or SPE techniques. Due to its effect on enrichment and purification with reduced matrix interference, SPE is often used as a complex matrix sample preparation method. In order to reduce or eliminate matrix effects, many different types of adsorbents have been used during SPE cleanup, such as TPT, PSA, GCB, and aminopropyl silylated silica gel, with one or more of these adsorbents [24,25,26,27]. Although using SPE columns for purification consumed more time, the matrix effect was lower than QuEChERS. Over 300 pesticides were detected in tea using GCB/PSA mixed-mode solid-phase extraction cartridges [24] or new multilayer solid-phase extraction cartridges [27].

In terms of detection technology, high-throughput screening of trace pesticides is necessary to ensure the safety of tea and reduce the economic losses due to pesticide residues. Gas chromatography or liquid chromatography was combined with low-resolution mass spectrometry, such as GC-MS, GC-MS/MS, LC-MS, and LC-MS/MS, to achieve the multi-residue detection of 400 and 653 pesticides in tea samples [21,23]. However, these methods may obtain false-positive or -negative results due to interference ions and low resolution. Compared with low-resolution mass spectrometry, high-resolution mass spectrometry (HRMS) technology has absolute advantages in screening and identifying compounds, such as a higher sensitivity and higher mass accuracy, and the detection is not limited by the number of targets [28,29,30,31,32]. Therefore, it is necessary to establish a multi-class method for the screening and determination of pesticide residues in tea using HRMS.

Bearing in mind the advantages provided by HRMS in terms of the sensitivity, number of compounds determined, and precision, the objective of this work was the development of a multi-class methodology to determine and quantify more than 470 compounds (including pesticides and their metabolites) in green tea. For that purpose, an SPE extraction procedure was tested for sample preparation, and LC–QTOF-MS was used to separate and quantify the target compounds, increasing the sample throughput. A self-built 479 pesticides mass spectral library was evaluated to increase the accuracy of pesticide residue identification. All Ions MS/MS acquisition mode was optimized to improve the sensitivity and accuracy of pesticide detection. The performance of the proposed method was evaluated on the linearity, SDL, LOQ, recoveries, precision, and matrix effect (ME). Moreover, this method was employed to screen pesticide residues in 95 commercially available green tea samples to demonstrate its applicability.

## 2. Materials and Methods

### 2.1. Reagents and Apparatus

All pesticide standards (purity grade, >98%) were obtained from Tianjin Alta Company (Tianjin, China); mass spectrometry grade formic acid and ammonium acetate; HPLC grade toluene, acetonitrile, and methanol were purchased from Honeywell Company (Morris Plains, NJ, USA); anhydrous Na_2_SO_4_, acetic acid, anhydrous MgSO_4_, and sodium chloride were analytical grade (Anpu Company, Shanghai, China); TPT cartridge (Bona Agel Technology Co., Ltd., Tianjin, China). LC-QTOF-MS was assembled with Agilent 1290 Infinity II LC and Agilent 6545 QTOF mass spectrometer which was configured with Agilent Dual Jet Stream ESI ionization source (Santa Clara, CA, USA). A vortex mixer (AS ONE, Osaka, Japan), SR-2DS oscillator (Taitec Corporation, Tokyo, Japan), solid-phase extraction, and parallel vacuum concentrator were purchased from Raykol Scientific Instruments (Xiamen, China), and a nitrogen evaporator (EVAP 112; Organomation Associates, Berlin, MA, USA) was also used. Deionized water was obtained via a Milli-Q high-purity water generator from Milford Company (Boston, MA, USA).

### 2.2. Standard Solution Preparation

Individual stock standard solutions of each pesticide (1 g/L) were prepared in methanol or other appropriate reagents depending on their solubility, which was stored at 4 °C away from light. Working standard solutions (10 mg/L) were prepared by mixing the stock standard solutions and the serial dilution solutions with methanol. The mixed standard solution was prepared at the appropriate concentration according to the experiment’s needs. Matrix-matched standard solutions were prepared by adding an adequate volume of working standard solution into the vial and then drying the solution under nitrogen. The residue was redissolved in 1 mL of the blank extractions.

For HRMS to function optimally, the instrument must be properly calibrated and maintained. Preparation of high-resolution mass spectrometer tuning solution: 10 mL of the tuning solution transferred into 100 mL vial, 88.5 mL of acetonitrile, 1.5 mL of ultrapure water, and 5 μL of HP-0321 (Agilent Company, Santa Clara, CA, USA) were then added into the vial. The resultant diluted tunning solution was thoroughly mixed via ultrasonication before being used for mass spectrometer tunning.

### 2.3. Samples Collection and Preparation

This study was performed with 95 green tea samples collected in China, including 40 samples obtained from tea plantations in different provinces and 55 samples bought from supermarkets. In the supermarket samples, 30 samples were from the current year’s domestic production, and 15 were from the previous year’s tea samples. The tea samples were ground, homogenized, sieved through the 18-mesh sieve, and stored in a refrigerator at 4 °C. All these samples were prepared and analyzed using the developed method, and the results of free of pesticides were used as blank matrixes for preparing the standard curve and the recovery studies.

Samples were prepared via a modified SPE method described by Pang et al. [21]. The SPE method proved to be suitable for pesticide multi-residue analysis in tea. The pretreatment procedure entailed the following steps: 

Extraction: 4 g of the dried tea sample was accurately weighed out and transferred to an 80 mL test tube. Then, the dried tea sample was hydrated with 3 mL water for 5 min followed by extraction using 30 mL acetonitrile. After extraction, the test tube was homogenized for 1 min at 13,500 r/min, then 2 g NaCl was added into the test tube, which was homogenized for 1 min and centrifuged at 4200 r/min for 5 min sequentially. An amount of 15 mL of the extracted sample supernatant was then transferred into the collection tube and was concentrated to 2 mL via parallel evaporation at 37 °C in a water bath and 120 mbar pressure before purification. 

Cleanup: About 1.5 cm height of anhydrous Na_2_SO_4_ was added in the SPE column cartridge, which was then activated using 4 mL of acetonitrile-toluene (3:1, *v*/*v*) with the effluent discarded. After the column activation, the sample solution was loaded into the SPE cartridge, and all the eluent was collected. The eluent was then concentrated to about 0.5 mL at 37 °C in a parallel concentrator and further dried under nitrogen. The resultant residue was redissolved in 1 mL of acetonitrile aqueous solution (3:2, *v*/*v*), mixed thoroughly under ultrasonication, and filtered through 0.22 μm nylon membrane for LC-QTOF-MS analysis.

### 2.4. Establishment of Compound Database

Individual pesticide standard solution (200 µg/L) was prepared using the above-stored pesticide stock solution. LC-QTOF-MS was used to collect data in Full-Scan and Targeted MS/MS mode under the optimal chromatographic and mass spectrometry conditions. In Full-Scan mode, the molecular formula, adduct ions, retention time, and other information of all pesticides were collected and imported into Agilent PCDL management software (version B.07.00) to form a primary accurate mass database. Based on the above pesticide results, targeted MS/MS mode was applied to acquire the secondary mass spectra of each individual pesticide compound, and the resultant spectra were then imported into the primary accurate mass database and formed the lab-customized PCDL containing secondary mass spectra information (an accurate mass database with a secondary mass spectrum library). Finally, the lab-customized PCDL was used to validate the screening results qualitatively.

### 2.5. LC-QTOF-MS Parameters

The separation was performed on a ZORBAX SB-C18 column (100 mm × 2.1 mm, 3.5 μm, Agilent, CA, USA); 5 mmol/L ammonium acetate aqueous solution and formic acid acetonitrile (1/1000, *v*/*v*) solution were used as mobile phase A and mobile phase B, respectively. The gradient program was set as follows: 0 min, 99% A; 3 min, 70% A; 6 min, 60% A; 9 min, 60% A; 15 min, 40% A; 19 min, 10% A; 23 min, 10% A; 23.01 min, 99% A. The post-run equilibrium time was 4 min. The column temperature was set at 40 °C and the flow rate was 0.4 mL/min. The injection volume was 5 μL.

Dual Agilent Jet Stream (AJS) ESI source (CA, USA) was operated under positive ionization mode being set; nitrogen was used as nebulizer gas at 0.14 MPa; capillary voltage was set at 4 kV; the drying gas temperature was set at 325 °C with a flow rate of 12.0 L/min; sheath gas temperature was set at 375 °C with flow rate 11.0 L/min; fragmentation voltage: 145 V. All Ions MS/MS mode parameter settings: acquisition range was *m*/*z* 50–1000, data acquisition rate was 4 spectra/s; collision energy was 0 eV at 0 min, and collision energy was set to 0, 15, and 35 eV in consecutive order after 0.5 min.

### 2.6. Method Validation

The methodology was validated in order to ensure that the obtained results were reliable. The parameters were chosen keeping in mind international guidelines [33]. The parameters tested were selectivity, screening detection limit, limit of quantification, linearity, matrix effect, recovery, precision, and uncertainty. To define the screening detection limit (SDL), refer to the European SANTE/11312/2021 guidelines. SDL is the lowest level at which an analyte has been detected with an acceptable false-negative rate of ≤5%. LOQs were defined as the lowest spiked concentration which satisfies the accepted recovery and precision (within 70–120% and less than 20%, respectively). Calibration curves were investigated with a series of standard solutions at concentration levels of 1 × LOQ, 2 × LOQ, 5 × LOQ, 10 × LOQ, and 20 × LOQ, for each pesticide in the blank sample. Matrix effects (ME) were evaluated by comparing the signal intensity of the matrix standard with the pure solvent standard at the same concentration. Recovery and precision were determined based on 3 spiked levels at 1 × LOQ, 2 × LOQ, and 10 × LOQ with 5 replicates at each spiked level [34]. 

Agilent Mass Hunter (version B. 08.00) software was used to analyze the data with the lab-customized pesticide PCDL as the compound source. The data results were analyzed and summarized by Excel (Version. 2016) software.

## 3. Results and Discussion

### 3.1. Establishment of Compound Database

**Figure 1:**
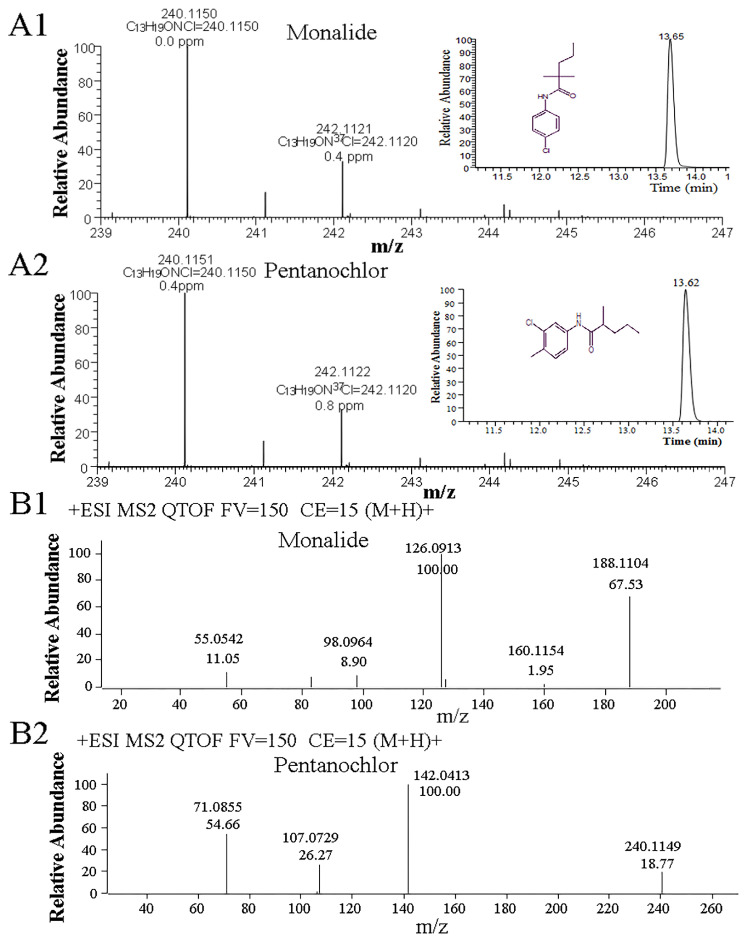
(**A**): the MS^1^ and extracted ion chromatogram of monalide and pentanochlor (**A1**,**A2**); (**B**): the MS^2^ of monalide and pentanochlor (**B1**,**B2**).

The high-resolution mass spectrometry database is the basis for the LC-QTOF-MS qualitative and quantitative analysis. Although commercial companies provide databases of the corresponding pesticide compounds, there are false-positive or false-negative test results during practical application. Therefore, to improve the accuracy and reliability of the method identification, this study decided to build an in-house pesticide database. Firstly, individual standard solutions of 200 μg/L for the investigated pesticides were injected into LC-QTOF-MS, working in MS mode (collision voltage was set to 0 V), and the ion source was set to positive. The precursor ion form, accurate mass, and retention time of the target compound were obtained in this mode. However, it was difficult to accurately identify some isomers based on the above mass spectrometry information (MS^1^). As shown in Figure 1A, monalide and pentanochlor have the same molecular formula C_13_H_18_ClNO, their adduct ion forms were [M + H]^+^, and their exact theoretical masses were 240.1150. The retention time deviation of the two compounds was less than 0.1 min. Therefore, the two pesticides cannot be accurately distinguished from the MS^1^ information mentioned above alone. Hence, acquiring secondary mass spectrometry information (MS^2^) is essential to enhance the qualitative identification confidence of the compounds.

Based on the MS^1^ information of the target compounds, the MS^2^ information was collected in Targeted MS/MS mode to build a lab-customized personal compounds database and library (abbreviated as PCDL) containing primary accurate mass data and secondary mass spectra information. The MS^2^ information in the PCDL is crucial for the identification of compounds. Taking the pair of isomers mentioned above, monalide and pentanochlor, as an example, as shown in Figure 1B, monalide was fragmented into two characteristic product ions with an *m*/*z* of 188.1104 and 126.0913. In comparison, two specific fragmented product ions with an *m*/*z* of 142.0413 and 107.0729 were obtained for the pentanochlor protonated molecular ion; hence, the two pesticides could be confidently identified based on the MS^2^ information. In the established PCDL, some isomer pesticides were similar to those above, such as pebulate, vernolate, etc. Therefore, MS^2^ was crucial for identifying the compounds with the same molecular weight and chemical formula. The established high-resolution PCDL contains 479 pesticides with an accurate mass, retention time, and product ion (see Table S1). To better utilize the PCDL for accurately identifying pesticide compounds, the data collected by LC-QTOF-MS should contain both MS^1^ and MS^2^ information.

### 3.2. Development of All Ions MS/MS Method

In the traditional LC-Q-TOF acquisition mode, we usually used Full Mass mode to collect the MS^1^ information and Target MS/MS mode to collect the MS^2^ information [27]. In this study, we utilized All Ions mode to simplify the experimental process, simplifying two injections into a single one and collecting the information of the addition and fragment ions simultaneously. On the basis of ensuring the accuracy, stability, and reliability of the results, we effectively reduced the running time of the instrument and doubled the whole experimental efficiency. The identification and quantification of target compounds were performed in All Ions MS/MS acquisition mode by LC-QTOF-MS. All Ions MS/MS mode has been reported for the qualitative screening of target analytes in the complex matrix [35]. In this study, the identification of pesticides in tea was carried out by All Ions MS/MS acquisition mode, and the data were collected from the three-channel collision energy of 0, 15, and 35 eV (Figure S1). Complete data acquisition in this mode requires alternating the three collision energy channels mentioned above. Nonetheless, sufficient scan points can be obtained to meet the quantitative analysis requirements of the compounds when the probable acquisition rate was selected. By ramping the acquisition rate, it was found that the 4 spectra/s acquisition rate can provide 10–30 data points per channel, which is sufficient to meet both the qualitative and quantitative needs.

**Figure 2:**
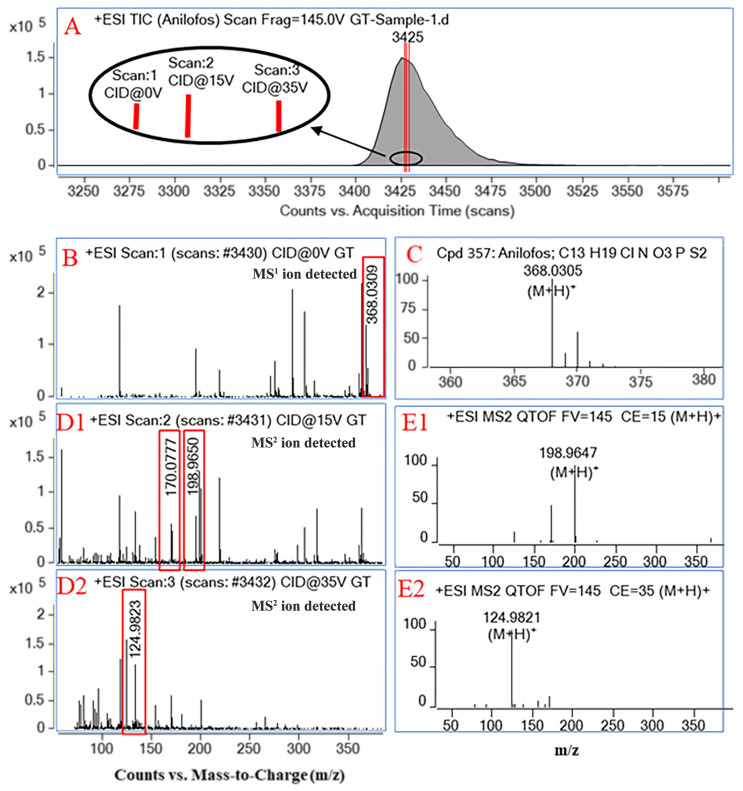
(**A**): TIC of anilofos in All Ions MS/MS mode; (**B**,**D1**,**D2**): All Ions MS/MS acquisition mode obtains mass spectrum information at 0, 15, and 35 eV three-channel collisionS energy, respectively; (**C**,**E1**,**E2**): mass spectra of anilofos standard products in the lab-customized PCDL at collision energies of 0, 15, and 35 eV, respectively.

As shown in Figure 2A, 75 data points were collected for the target pesticide anilofos in All Ions MS/MS acquisition mode, averaging 25 data points per channel. The mass spectrum information of the 0 eV collision energy channel shown in Figure 2B is consistent with the MS^1^ information of anilofos in the customized PCDL, as shown in Figure 2C. The fragmentation ion mass spectrum information of the 15 eV or 35 eV channel collision energy is also consistent with the mass spectrum information generated under the corresponding collision energy of anilofos in the PCDL, as shown in Figure 2D or Figure 2E. Therefore, All Ions MS/MS was performed with the 0 eV collision energy channel for identification and quantification, and the 15 eV or 35 eV collision energy channel were complementary for the identification of the compounds. This study demonstrated the unique advantage of integrating MS^1^ and MS^2^ scans in high-resolution All Ions MS/MS assays on a QTOF platform with multiple pesticide residue qualification.

### 3.3. Optimization in Sample Preparation

Selecting the appropriate extraction solvent is particularly important to improve the extraction efficiency and reduce matrix interference. Acetonitrile has been reported to have a wide range of polar solubility compared to other solvents, which can effectively reduce lipid/pigment co-extraction in the extraction process, significantly reduce the interference of matrix components, and achieve the satisfactory recovery of multiple pesticide residues [21]. Owing to the dry nature of tea samples, some studies reported the scheme of tea soaking with water followed by organic solvent extraction [5,22,36], while other studies adopted the direct addition of an organic solvent for homogeneous extraction [21,23]. To analyze multi-pesticide residue in green tea using LC-QTOF-MS, SPE was applied and the extraction of pesticides investigated with and without hydration. The comparative experimental study revealed that the recovery of pesticides investigated and studied in the experimental group with water hydration (298 pesticides in total, added at a concentration of 10 µg/kg) was significantly higher than that of the control group without water hydration. For example, the percentage of pesticides that met the SANTE/11312/2021 technical requirements Rec & RSD (70% ≤ Rec ≤ 120%, RSD ≤ 20%) in the hydration group using 3 mL of water before extraction was 28.9% (86/298) higher than that of the experimental group that was extracted directly without water soaking. This was sufficient to indicate that hydration with water enhanced the ability of acetonitrile to penetrate into tea tissue in the subsequent extraction process and improved the extraction efficiency of pesticides. Therefore, in this study, the extraction was performed after water soaking.

**Figure 3:**
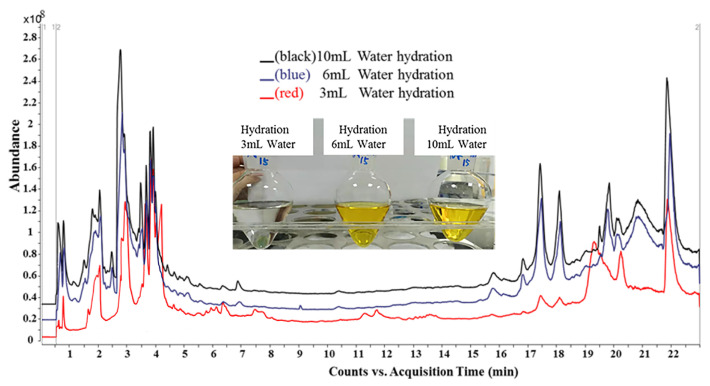
Comparison of collected solution and corresponding total ion chromatogram of tea samples hydrated with different volumes of water (3 mL, 6 mL, and 10 mL).

The optimization of hydration parameters in this investigation is based on the literature research, such as the amount of water added to the tea leaves ranging from 1–10 mL/g and the hydration time ranging from 5–30 min [5,22]. In the present study, tea was hydrated with 3 mL, 6 mL, and 10 mL of water, respectively. As shown in Figure 3, when the hydration volume gradually increased, the color of the extract in the pear-shaped bottle also deepened, which inevitably resulted in the increase in the co-extract interferents, which could post a negative effect on the target measuring accuracy and instrument performance. As illustrated by the TIC in Figure 3, the baseline noise and interference peaks increased with the increasing hydration volume. Therefore, 3 mL of water was selected for hydration. For the optimization of the hydration time, there was no significant change in the recovery of the pesticides under 5, 10, 20, and 30 min. Instead, the increase in the hydration time deepened the color of the extract and increased the overall pretreatment time, so 5 min was selected as the hydration time.

Notably, after hydration, the overall results were significantly better than those of the non-hydrated control group (28.9%). However, some water-soluble tea components, such as theophylline and tea polyphenols, were also extracted, which enhanced the matrix background interference. By comparison, it was found that the recovery of ditalimfos, ethoxyquin, pyridalyl, and thifluzamide was significantly higher without hydration. On the other hand, the detection of some pesticides was seriously affected by hydration due to matrix interference. For example, as shown in Figure S2, phorate was not identified in the experimental hydration group at a 10 µg/kg spiked concentration. The SDL of phorate in the hydrated group was 50 µg/kg, while the SDL in the non-hydrated control group was 10 µg/kg, which also suggested the difference of co-extract interferents under hydration and dry conditions. Therefore, to reduce the effect of matrix interference, subsequent experiments were conducted for optimizing the sample purification procedure by selecting SPE columns.

**Figure 4:**
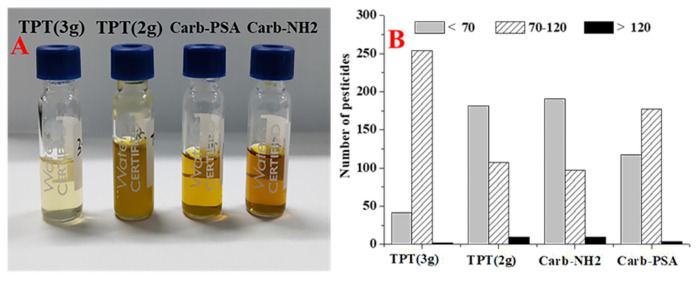
(**A**): Comparison of purification effects of different SPE cartridges; (**B**): comparison of the number of pesticides detected under different SPE cartridge purification (spiked with 20 µg/kg, n = 5).

In order to reduce the interference of co-extracts on the identification of target compounds, the SPE cartridges with different adsorption materials such as Carb-NH_2_, Carb-PSA, Cleanert TPT (2 g), and Cleanert TPT (3 g) were compared. In terms of co-extract removal, Carb-NH_2_, Carb-PSA, and Cleanert TPT were comparable in terms of the purification effect. However, with the increasing TPT dosage, the purification effect was significantly improved, as shown in Figure 4A. The purification materials used in the solid-phase extraction process can adsorb the interfering substances in the extraction solution and adsorb the target pesticide compounds. Therefore, the recovery experiments were carried out to determine the effect of different purification materials, as shown in Figure 4B. The results showed that the recovery of the Cleanert TPT (3 g) cartridge and Carb-PSA cartridge was higher than that of the Carb-NH2 cartridge and Cleanert TPT (2 g) cartridge. Considering the purification effect, the Cleanert TPT (2 g) and Carb-NH_2_ cartridges were weak in removing pigments and co-extracts, and the overall recovery of the added experimental pesticides was poor. Carb-PSA cartridges with double NH2 functional group packing can remove polar interferents such as organic acids and pigments from tea well, but the qualified recovery of pesticides was less than 60.0%. The most outstanding results were obtained for the Cleanert TPT (3 g) cartridge with 298 pesticides spiked (20 µg/kg), and the recovery rate was 70.0–120% for 254 species, which accounted for 85.2% of the total spiked pesticides. Therefore, according to the purification effect and recovery distribution, Cleanert TPT (3 g) was selected as the final solid-phase extraction purification cartridge. 

Tea samples were hydrated with 3 mL of water and extracted with acetonitrile, then purified with a TPT column. The effectiveness of this combination will be discussed further under “Matrix effect”. This sample preparation technique is suitable for analyzing multi-pesticide residues in green tea. 

### 3.4. Matrix Effect

Impurities such as fats, lipids, and polyphenols in tea can inhibit or enhance the response to pesticides. The matrix effect is calculated as follows [37]:

(1) $$ME\;(\%)=\left(\frac{peak\;area\;of\;the\;matrix-matched\;standard}{peak\;area\;of\;the\;solvent\;standard}-1\right)\times 100,$$

**Figure 5:**
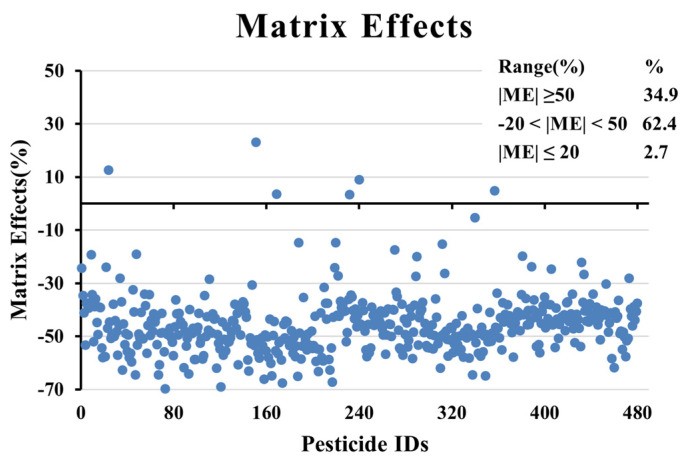
Distribution of matrix effects of 479 pesticides in green tea analysis method: Strong matrix effect: |ME| ≥ 50, Medium matrix effect: 20 < |ME| < 50, Weak matrix effect: |ME| ≤ 20.

In order to evaluate the matrix effect, 50 μg/kg of pesticide standard was spiked into tea matrices. The matrix effect is classified into three groups based on its intensity (Strong matrix effect: |ME| ≥ 50; Medium matrix effect: 20 < |ME| < 50; and Weak matrix effect: |ME| ≤ 20). The results are shown in Figure 5. Signal suppression was typically observed. It can be found that 13 compounds (2.7%) met that criterion (|ME| ≤ 20), meaning pesticides could be quantified using solvent calibration curves. The matrix effect beyond ±20% can be overcome by dilution, standard addition, isotope internal standard addition, matrix-matched calibration curve, and other approaches [38]. Moreover, out of 479 pesticide residues, 299 and 167 pesticides showed moderate and strong matrix effects, respectively. The result showed that the matrix effect was stronger for most pesticides (97.3%), which may affect the mass accuracy and quantitation of analytes in the samples. It was enough to explain that tea as a complex matrix sample had strong matrix background interference. Therefore, a matrix-matched standard calibration curve was used to reduce quantitation bias due to the matrix effect.

### 3.5. Method Validation

#### 3.5.1. Selectivity, Screening Detection Limit, Limit of Quantification, and Linear Range

The benefit of acquiring fragment ions by All Ions MS/MS instead of Full Mass + Targeted MS/MS is illustrated in Figure 2. Although it greatly reduces time, it shows adequate selectivity. For example, as shown in Figure S3, matrix interference was well avoided in napropamide at low concentration levels of 10 μg/kg and 1 μg/kg. Meanwhile, the two pesticides, cycloate and pyridaben, also shown in Figure S3, can still achieve accurate identification under the interference of the tea matrix. The identification of any pesticides using LC-QTOF-MS was based on the mass accuracy and chromatographic retention time. SANTE/11312/2021 required ≥2 diagnostic ions (preferably the precursor ion and its product ion) with a mass accuracy of <5 ppm along with a retention time tolerance of ±0.1 min for identification.

**Figure 6:**
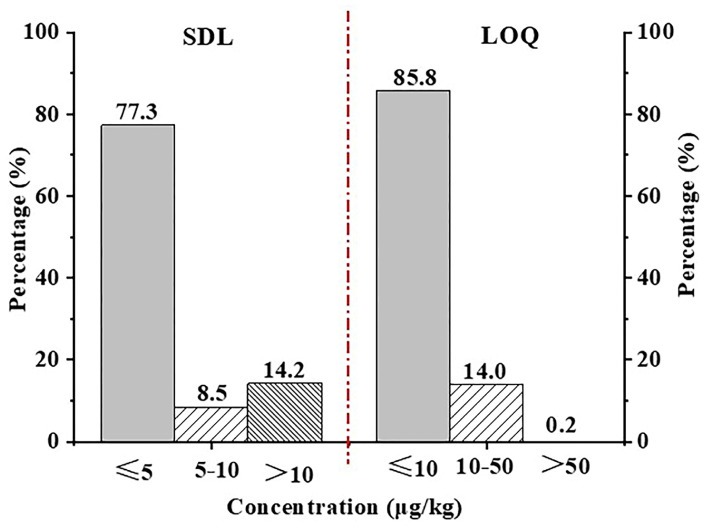
Distribution of SDL and LOQ of 479 pesticides in tea.

The SDL refers to the requirements of the SANTE/11312/2021 guidance document, and the relevant experiments are performed as described in Section 2.6. The SDLs of 479 pesticides in tea (1–50 μg/kg), of which the SDLs of 372 pesticides are less than or equal to 5 μg/kg, accounted for 77.3%. The specific SDL distribution is shown in Figure 6. The results indicated that the method has a high screening sensitivity (≤10 μg/kg, 85.8%). 

A series of decreasing concentrations of matrix-matched pesticide standard solutions were tested and the levels which produced chromatographic peaks with S/N = 10 for the quantifier ions were set as the LOQs of the corresponding compounds in the method. The LOQ of 479 pesticides in green tea ranged from 5–55 μg/kg, of which 408 were not more than 10 μg/kg, accounting for 84.8% (see Table S1). The specific LOQ distribution is shown in Figure 6. It can be seen from the figure that this method has a good quantitative ability at a low concentration level (≤10 μg/kg, 84.8%).

The linear range of 1–20 × LOQ in green tea was investigated. The linear equation was acceptable when the coefficient of determination (R^2^) was ≥0.9806. The results are shown in Table S1. All pesticides showed good linearity in their respective linear ranges, with R^2^ meeting the established criteria.

#### 3.5.2. Recovery and Precision

**Figure 7:**
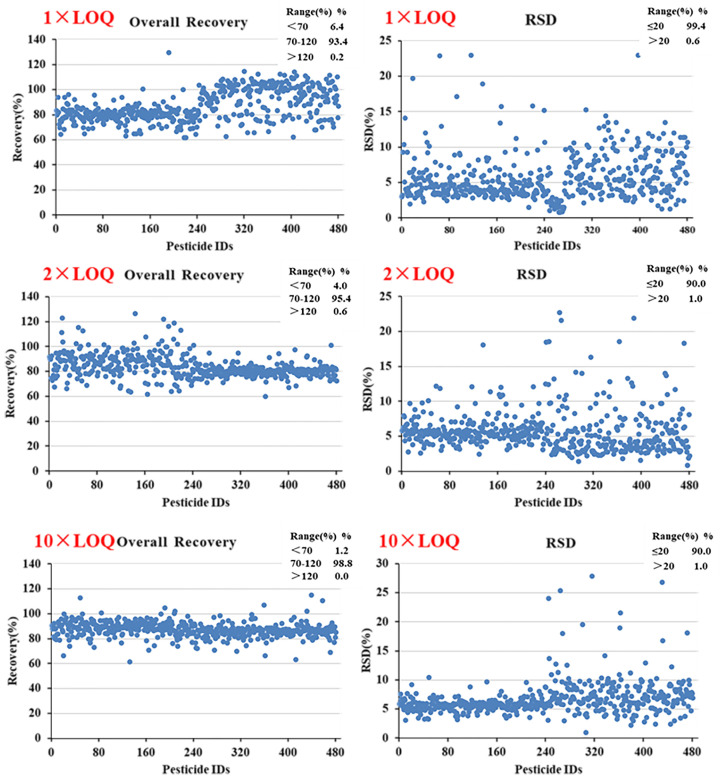
Recovery rate and precision of 479 pesticides in tea at three levels (1 × LOQ, 2 × LOQ, and 10 × LOQ.

To investigate the accuracy and precision of the method, blank green tea samples were spiked at 1 × LOQ, 2 × LOQ, and 10 × LOQ (six samples for each level) prior to analysis. The method performance results are shown in Figure 7. 

At the three spiked levels, 92.7, 94.6, and 97.7% of the detectable pesticides in green tea were recovered within the range of the EU guidelines for pesticide residue analysis, i.e., between 70 and 120% with a precision of ≤20%. For some pesticides, such as kadethrin and oxydemeton-methyl, the recovery was below 70% due to the loss in the sample extraction process or adsorption by the purification materials. Nonetheless, the proposed method meets the performance requirements and is suitable for the routine analysis of multiple pesticide residues in green tea.

#### 3.5.3. Measurement Uncertainty

Measurement uncertainty is an important factor in evaluating the performance of a method. For the calculation of uncertainty, the approach described in the Codex Alimentarius guideline was followed [39]. The uncertainty was expressed as a percentage (%U) and the results summarized in Table S1. According to the results, for green tea, 58.5% of pesticides yielded a measurement uncertainty of 20.0% or less, 32.2% of pesticides yielded a measurement uncertainty between 20.1 and 30.0%, 5.2% of pesticides yielded a measurement uncertainty between 30.1 and 40.0%, 2.3% of pesticides yielded a measurement uncertainty between 40.1 and 50.0%, and only 1.9% of pesticides yielded a measurement uncertainty greater than 50.0%. Based on the above results, 98.1% of the pesticides agreed with the expanded uncertainty value established by the European Reference Laboratories (≤50%) in their interlaboratory test studies. That means that the uncertainty of the method is adequate for this application.

### 3.6. Analysis of Actual Samples

**Figure 8:**
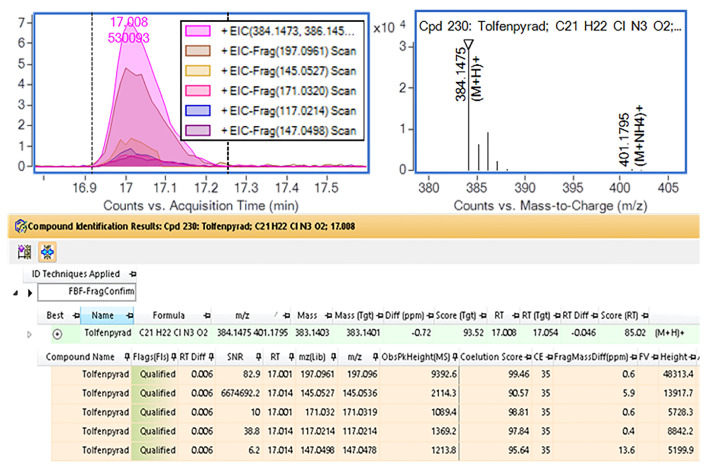
Qualitative screening results of tolfenpyrad in green tea samples.

A total of 95 green tea samples were purchased from the market. Samples were prepared under the above conditions. Among them, 89.5% (85 cases) of the samples were detected with pesticide residues involving 47 kinds of pesticides. According to the maximum allowable limits in tea specified in GB 2763-2021 [9], the detected levels of pesticide residues in the above samples were all below the corresponding maximum allowable limits. The results showed that the residues of tolfenpyrad, buprofezin, and pyridaben were the most prominent in 95 tea samples, and the occurrence frequency of pesticides was more than half of the number of tea samples, 75, 59, and 54 times, respectively. The screening results of tolfenpyrad (insecticide) in the actual samples are shown in Figure 8. The MRL of this pesticide in tea specified in GB 2763-2021 of China is 50 mg/kg, which is relatively high due to the relatively low risk of toxicity. However, such a high occurrence frequency (75/95) of pesticides in tea and their relatively high residue levels (1 mg/kg residue levels above the sample reached six cases) may also cause certain safety risks when under long-term exposure.

In addition, the specified MRL values of certain pesticide residues vary significantly among different countries or regions. For example, regarding the EU MRL requirements for tea, up to 24 batches of tea samples with pesticide residues exceed the EU MRL requirements, which would impact the international tea trade, as shown in Table 1. 

Therefore, it is necessary to develop analytical techniques with high-throughput and high sensitivity to meet the requirements of the corresponding MRL values. The high-throughput screening and quantitative methods for 479 pesticide chemical pollutants in green tea based on solid-phase extraction–liquid chromatography time-of-flight mass spectrometry developed in this study have filled the corresponding gap well.

## 4. Conclusions

The present method consists of solid-phase extraction and a simultaneous screening and confirmatory analysis with LC-QTOF-MS using the mode of All Ions MS/MS, which shows a good quantitative and qualitative ability at the same time. It is also a highly efficient analytical method with advantages of saving time and costs on sample analysis. The method has been validated for achieving 479 pesticides in green tea. After applying it to the tea samples obtained from tea plantations and supermarkets, the high-occurrence frequency of tolfenpyrad, buprofezin, and pyridaben was found. Compared to the results from the literature, some contaminants need to receive more attention regarding their monitoring and control. LC-QTOF-MS along with SPE offers a practical tool for pesticide residue analysis in routine monitoring programs for food safety.

## Figures and Tables

**Table 1:** Pesticide, concentration range, LOQ, EU-MRL, China-MRL, and detection frequencies of samples exceeding EU-MRL.

Pesticide	Concentration Range (mg/kg)	LOQ (mg/kg)	EU-MRL (mg/kg)	China-MRL (mg/kg)	Exceeding MRL Samples	
					Number	%
Buprofezin	0.138–0.529	0.005	0.05	10	5	5.3
Pyridaben	0.125	0.005	0.05	5	1	1.1
Abamectin	0.262	0.005	0.05	/	1	1.1
Chlorpyrifos	0.046–0.731	0.005	0.01	2	7	7.4
Triadimenol	0.223–1.400	0.005	0.05	/	8	8.4
Propiconazole	0.064	0.005	0.05	/	1	1.1
Chlorantraniliprole	0.106	0.005	0.02	/	1	1.1

## Data Availability

The data used to support the findings of this study can be made available by the corresponding author upon request.

## Supplementary Materials

The following supporting information can be downloaded at: https://www.mdpi.com/article/10.3390/foods12224177/s1, Figure S1: All Ions MS/MS data acquisition mode parameter settings; Figure S2: phorate qualitative screening match results in tea samples of the hydration experiment group (sample spiked concentration 10 µg/kg); Figure S3: (A): sample spiked napropamide concentration 10 µg/kg; (B) sample spiked napropamide concentration 1 µg/kg; (C): sample spiked cycloate concentration 10 µg/kg; (D): sample spiked pyridaben concentration 10 µg/kg; Table S1: formula, linear ranges, the adduct ion, RT, SDL, LOQ, MS^1^, MS^2^, measurement uncertainty, and coefficient of determination (R^2^) of the 479 pesticides in tea.
