# Supplementary material for: Simultaneous Screening and Quantification of 479 Pesticides in Green Tea by LC-QTOF-MS
Source: Foods. 2023 Nov 20;12(22):4177. doi: 10.3390/foods12224177 (PMC10670754; doi:10.3390/foods12224177)
Supplement: Supplementary file 1 [file foods-12-04177-s001.zip › foods-2709671-supplementary.pdf]

Supplementary Material

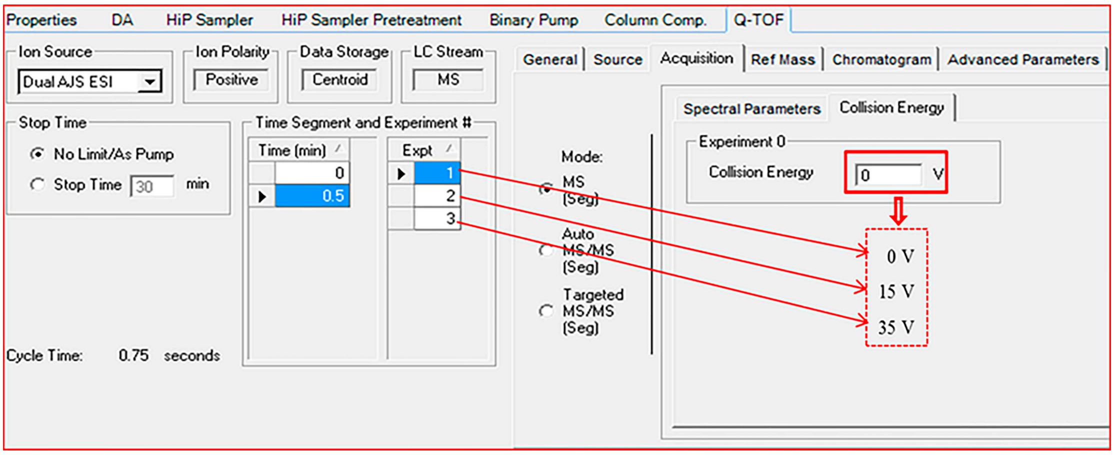

Figure S1. All Ions MS/MS data acquisition mode parameter settings.

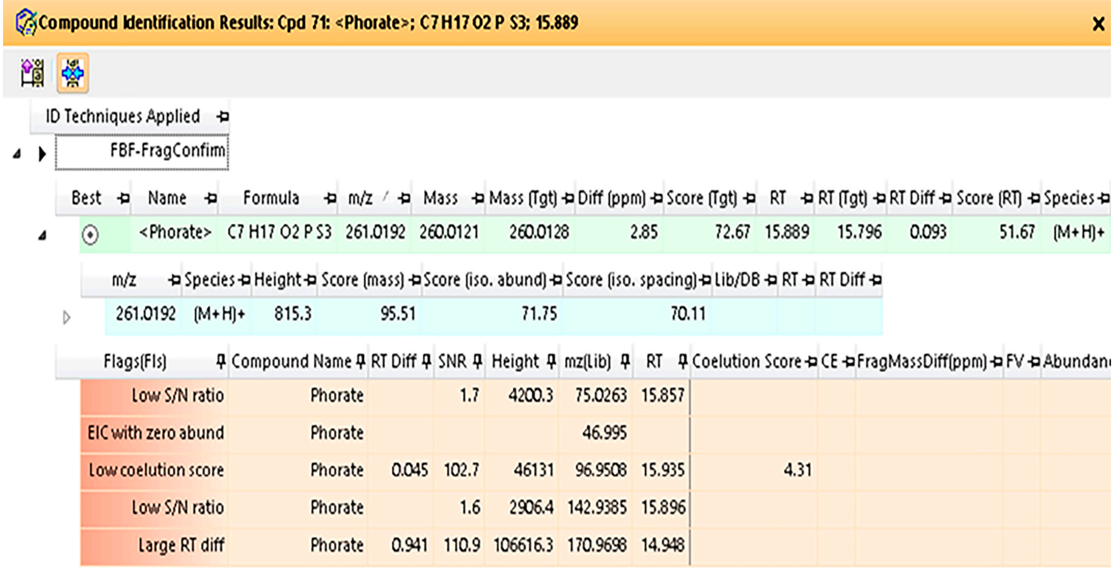

Figure S2. Phorate qualitative screening match results in tea samples of the hydration experiment group (Sample spiked concentration 10 μg/kg).

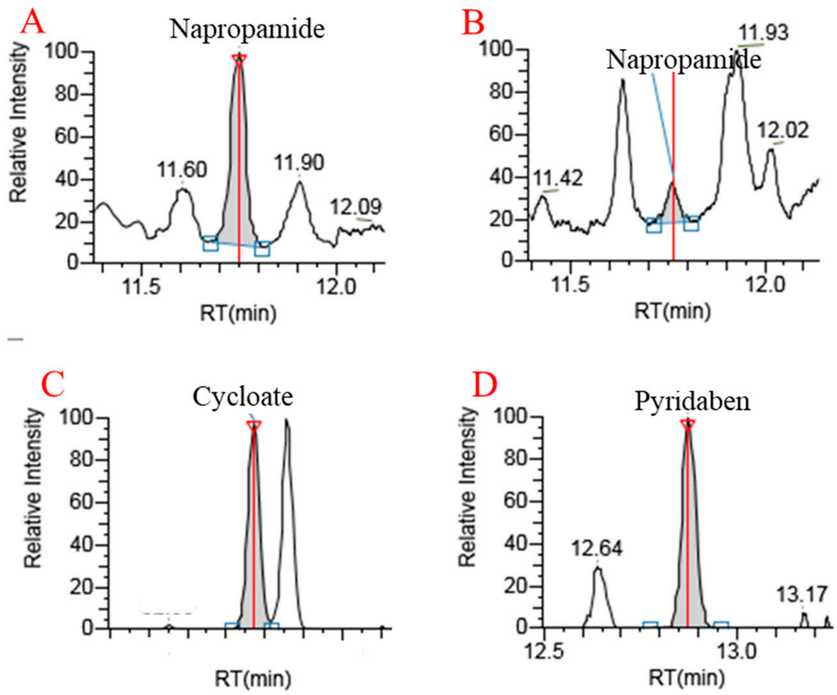

Figure S3. A:Sample spiked napropamide concentration 10 μg/kg; B Sample spiked napropamide concentration 1 μg/kg: C: Sample spiked cycloate concentration 10 μg/kg D: Sample spiked pyridaben concentration 10 μg/kg.

Table S1. Formula, linear ranges, the adduct ion, RT, SDL, LOQ, MS[1], MS[2], Measurement uncertainty and coefficient of determination (R^2) of the 479 pesticides in tea.

NO.	Compound	Formula	Adduct ion (m/z)	RT/min	Quantitative ion (m/z)	Product ion (m/z)	LOQ (μg/kg)	Linearity (μg/kg)	R^2	Measurement uncertainty (%)
1	2.3.5-Trimethacarb	$C_{11}H_{15}NO_2$	$[M+H]^+$	7.09	194.1176	122.0726	5	5-100	0.9912	19.3
2	3,4,5-Trimethacarb	$C_{11}H_{15}NO_2$	$[M+H]^+$	7.37	194.1163	137.0961	5	5-100	0.9982	24.4
3	Acibenzolar-S-methyl	$C_8H_6N_2OS_2$	$[M+H]^+$	9.06	210.9994	136.009	5	5-100	0.9987	54.5
4	Akton	$C_{12}H_{14}Cl_3O_3PS$	$[M+H]^+$	18.03	374.9539	96.9508	5	5-100	0.9844	21.8
5	Alachlor	$C_{14}H_{20}ClNO_2$	$[M+H]^+$	12.58	270.1255	238.0993	5	5-100	0.9995	25.8
6	Allidochlor	$C_8H_{12}ClNO$	$[M+H]^+$	5.07	174.068	98.0964	50	50-1000	0.9974	40.1
7	Ametoctradin	$C_{15}H_{25}N_5$	$[M+H]^+$	13.31	276.2183	176.0931	5	5-100	0.9989	14.4
8	Ametryn	$C_9H_{17}N_5S$	$[M+H]^+$	6.71	228.1277	68.0243	5	5-100	0.9983	31.8
9	Ancymidol	$C_{15}H_{16}N_2O_2$	$[M+H]^+$	5.22	257.1285	81.0447	5	5-100	0.9836	13.8
10	Aramite	$C_{15}H_{23}ClO_4S$	$[M+NH_4]^+$	17.46	352.1347	191.143	25	25-500	0.9936	14.9
11	Aspon	$C_{12}H_{28}O_5P_2S_2$	$[M+H]^+$	19.03	379.0927	114.9613	5	5-100	0.9972	35.3
12	Athidathion	$C_8H_{15}N_2O_4PS_3$	$[M+H]^+$	13.46	331.0004	85.0396	5	5-100	0.9983	15.5
13	Atrazine	$C_8H_{14}ClN_5$	$[M+H]^+$	6.44	216.1011	174.0541	5	5-100	0.9979	30.4
14	Azaconazole	$C_{12}H_{11}Cl_2N_3O_2$	$[M+H]^+$	6.11	300.0301	158.9763	5	5-100	0.9973	26.6
15	azadirachtin	$C_{35}H_{44}O_{16}$	$[M+Na]^+$	6.47	743.2517	565.1704	5	5-100	0.9979	22.7
16	Azamethiphos	$C_9H_{10}ClN_2O_5PS$	$[M+Na]^+$	5.41	324.9809	111.9952	5	5-100	0.9975	15.2
17	Azinphos-ethyl	$C_{12}H_{16}N_3O_3PS_2$	$[M+H]^+$	13.4	346.0443	77.0389	5	5-100	0.9976	16.0
18	Beflubutamid	$C_{18}H_{17}F_4NO_2$	$[M+H]^+$	14.56	356.1268	91.0542	5	5-100	0.9977	18.4
19	Benazolin-ethyl	$C_{11}H_{10}ClNO_3S$	$[M+H]^+$	10	272.0143	169.9821	5	5-100	0.9894	24.1
20	Benoxacor	$C_{11}H_{11}Cl_2NO_2$	$[M+H]^+$	9.86	260.024	120.0444	50	50-1000	0.9971	47.5
21	Bensulfuron-methyl	$C_{16}H_{18}N_4O7S$	$[M+H]^+$	7.88	411.0969	149.0597	5	5-100	0.9821	24.5
22	Bensulide	$C_{14}H_{24}NO_4PS_3$	$[M+H]^+$	15.21	398.0678	313.9736	5	5-100	0.9979	41.5
23	Benthiavalicarb-Isopropyl	$C_{18}H_{24}FN_3O_3S$	$[M+H]^+$	9.39	382.1595	116.0706	5	5-100	0.9977	24.8
24	Benzoximate	$C_{18}H_{18}ClNO_5$	$[M+H]^+$	16.38	364.0946	105.0335	5	5-100	0.9979	20.1
25	Benzoylprop-Ethyl	$C_{18}H_{17}Cl_2NO_3$	$[M+H]^+$	15.36	366.0658	105.0335	5	5-100	0.9978	15.9
26	Bixafen	$C_{18}H_{12}Cl_2F_3N_3O$	$[M+H]^+$	13.55	414.0384	394.032	5	5-100	0.9979	21.1
27	Bromfenvinfos	$C_{12}H_{14}BrCl_2O_4P$	$[M+H]^+$	14.25	402.9263	98.9842	5	5-100	0.9984	26.0
28	Bromfenvinfos-Methyl	$C_{10}H_{10}BrCl_2O_4P$	$[M+H]^+$	11.02	374.895	127.0155	5	5-100	0.9967	27.2
29	Bupirimate	$C_{13}H_{24}N_4O_3S$	$[M+H]^+$	12.61	317.1642	44.0495	5	5-100	0.9982	53.8
30	Buprofezin	$C_{16}H_{23}N_3OS$	$[M+H]^+$	17.42	306.1635	57.0699	5	5-100	0.9983	27.3
31	Butachlor	$C_{17}H_{26}ClNO_2$	$[M+H]^+$	17.61	312.1725	57.0699	5	5-100	0.9966	38.3
32	Butralin	$C_{14}H_{21}N_3O_4$	$[M+H]^+$	18.28	296.1605	240.0979	5	5-100	0.9868	15.0
33	Buturon	$C_{12}H_{13}ClN_2O$	$[M+H]^+$	7.94	237.0789	53.0386	5	5-100	0.9825	16.8
34	Butylate	$C_{11}H_{23}NOS$	$[M+H]^+$	16.77	218.1573	57.0699	50	50-1000	0.9885	24.3
35	Carbofuran	$C_{12}H_{15}NO_3$	$[M+H]^+$	5.87	222.1125	123.0441	5	5-100	0.9954	14.9
36	Carbophenothion	$C_{11}H_{16}ClO_2PS_3$	$[M+H]^+$	18.27	342.9811	44.9793	25	25-500	0.9873	14.5

37	Carfentrazone-ethyl	$C_{15}H_{14}Cl_2F_3N_3O_3$	$[M+H]^+$	14.29	412.0435	345.9956	5	5-100	0.998	21.6
38	Carpropamid	$C_{15}H_{18}Cl_3NO$	$[M+H]^+$	14.77	334.0527	139.0309	5	5-100	0.9991	23.0
39	Chlorantraniliprole	$C_{18}H_{14}BrCl_2N_5O_2$	$[M+H]^+$	8.37	481.9781	283.9216	5	5-100	0.9957	11.9
40	Chlorbenzuron	$C_{14}H_{10}Cl_2N_2O_2$	$[M+H]^+$	12.86	309.0193	156.0211	5	5-100	0.9939	41.6
41	chlorotoluron	$C_{10}H_{13}ClN_2O$	$[M+H]^+$	6.15	213.0789	72.0449	5	5-100	0.9964	27.0
42	Chlorphoxim	$C_{12}H_{14}ClN_2O_3PS$	$[M+H]^+$	16.55	333.0224	96.9508	10	10-200	0.9984	21.7
43	Chlorpyrifos	$C_9H_{11}Cl_3NO_3PS$	$[M+H]^+$	17.83	349.9336	96.9508	5	5-100	0.9945	18.7
44	Chlorpyrifos-methyl	$C_7H_7Cl_3NO_3PS$	$[M+H]^+$	15.8	321.9023	124.9821	50	50-1000	0.9946	26.8
45	Chlorpyrifos-oxon	$C_9H_{11}Cl_3NO_4P$	$[M+H]^+$	11.87	333.9565	279.8909	5	5-100	0.9971	17.0
46	Chromafenozide	$C_{24}H_{30}N_2O_3$	$[M+H]^+$	13.12	395.2329	175.0754	5	5-100	0.995	20.4
47	Cinidon-Ethyl	$C_{19}H_{17}Cl_2NO_4$	$[M+H]^+$	17.04	394.0607	107.0491	50	50-1000	0.9965	15.3
48	Cinmethylin	$C_{18}H_{26}O2$	$[M+NH_4]^+$	17.26	292.227	105.0699	25	25-500	0.9877	10.4
49	Cinosulfuron	$C_{15}H_{19}N_5O_7S$	$[M+H]^+$	5.77	414.1078	183.0499	5	5-100	0.9835	13.3
50	Cloquintocet-mexyl	$C_{18}H_{22}ClNO_3$	$[M+H]^+$	16.91	336.1361	179.0128	5	5-100	0.998	40.9
51	coumaphos-oxon	$C_{14}H_{16}ClO_6P$	$[M+H]^+$	8.33	347.0442	211.0157	5	5-100	0.9946	22.7
52	Coumoxystrobin	$C_{26}H_{28}O_6$	$[M+H]^+$	17.45	437.1963	145.0648	5	5-100	0.9991	15.4
53	Crufomate	$C_{12}H_{19}ClNO_3P$	$[M+H]^+$	10.83	292.0864	108.0209	5	5-100	0.9991	17.8
54	Cumyluron	$C_{17}H_{19}ClN_2O$	$[M+H]^+$	10.96	303.1259	119.0855	5	5-100	0.9975	28.0
55	Cyantraniliprole	$C_{19}H_{14}BrClN_6O_2$	$[M+H]^+$	6.98	473.0123	285.9199	5	5-100	0.9985	20.3
56	Cycloate	$C_{11}H_{21}NOS$	$[M+H]^+$	15.51	216.1417	55.0542	5	5-100	0.9939	13.2
57	Cyclosulfamuron	$C_{17}H_{19}N_5O_6S$	$[M+H]^+$	12.39	422.1129	261.0288	5	5-100	0.9841	20.9
58	Cyflufenamid	$C_{20}H_{17}F_5N_2O_2$	$[M+H]^+$	16.6	413.1283	203.0227	5	5-100	0.9975	10.9
59	Cyprazine	$C_9H_{14}ClN_5$	$[M+H]^+$	6.45	228.1007	186.0541	5	5-100	0.9928	15.1
60	Cyprodinil	$C_{14}H_{15}N_3$	$[M+H]^+$	12.17	226.1339	93.0573	5	5-100	0.9963	16.5
61	Cyprofuram	$C_{14}H_{14}ClNO_3$	$[M+H]^+$	6.94	280.0735	69.034	5	5-100	0.9969	15.4
62	Daimuron	$C_{17}H_{20}N_2O$	$[M+H]^+$	11.17	269.1656	91.0542	5	5-100	0.9975	15.0
63	Desmetryn	$C_8H_{15}N_5S$	$[M+H]^+$	5.23	214.1121	172.0651	5	5-100	0.9977	17.8
64	Dialifos	$C_{14}H_{17}ClNO_4PS_2$	$[M+H]^+$	16.58	394.0098	181.0049	10	10-100	0.9918	37.9
65	Diazinon	$C_{12}H_{21}N_2O_3PS$	$[M+H]^+$	15.2	305.1083	96.9508	5	5-100	0.9968	16.5
66	Dibutyl succinate	$C_{12}H_{22}O_4$	$[M+H]^+$	14.22	231.1593	101.0233	25	25-500	0.9839	24.1
67	Dichlofenthion	$C_{10}H_{13}Cl_2O_3PS$	$[M+H]^+$	17.75	314.9773	96.9509	25	25-500	0.9992	10.9
68	Diclocymet	$C_{15}H_{18}Cl_2N_2O$	$[M+H]^+$	13.42	313.0869	172.9919	50	50-1000	0.9972	16.2
69	Diethatyl-Ethyl	$C_{16}H_{22}ClNO_3$	$[M+H]^+$	13.9	312.136	162.1277	5	5-100	0.997	29.0
70	Difenoconazole	$C_{19}H_{17}Cl_2N_3O_3$	$[M+H]^+$	14.73	406.072	251.0025	5	5-100	0.9982	12.0
71	Difenoxuron	$C_{16}H_{18}N_2O_3$	$[M+H]^+$	7.15	287.139	72.0444	5	5-100	0.9968	21.4
72	Diflubenzuron	$C_{14}H_9ClF_2N_2O_2$	$[M+H]^+$	12.2	311.0393	141.0146	50	50-1000	0.9894	29.3
73	Dimepiperate	$C_{15}H_{21}NOS$	$[M+H]^+$	16.14	264.1417	146.0634	10	10-100	0.9816	14.7
74	Dimethachlor	$C_{13}H_{18}ClNO_2$	$[M+H]^+$	7.74	256.1099	148.1121	5	50-100	0.9944	18.2
75	Dimethametryn	$C_{11}H_{21}N_5S$	$[M+H]^+$	11.38	256.159	186.0808	5	5-100	0.9963	16.8
76	Dimethenamid	$C_{12}H_{18}ClNO_2S$	$[M+H]^+$	9.81	276.082	244.0557	5	5-100	0.9966	16.8
77	Diphenamid	$C_{16}H_{17}NO$	$[M+H]^+$	7.95	240.1383	134.0964	5	5-100	0.9969	18.3
78	Dipropetryn	$C_{11}H_{21}N_5S$	$[M+H]^+$	11.42	256.159	102.0121	5	5-100	0.9961	23.5
79	Disulfoton sulfone	$C_8H_{19}O_4PS_3$	$[M+H]^+$	8.56	307.0253	96.9508	5	5-100	0.9995	18.0

80	Dithiopyr	$C_{15}H_{16}F_5NO_2S_2$	$[M+H]^+$	17.2	402.0618	248.0351	5	5-100	0.9975	15.2
81	Enestroburin	$C_{22}H_{22}ClNO_4$	$[M+H]^+$	16.79	400.1311	145.0648	5	5-100	0.9977	14.3
82	Etaconazole	$C_{14}H_{15}Cl_2N_3O_2$	$[M+H]^+$	11.07	328.0614	158.9745	5	5-100	0.999	15.5
83	Ethaboxam	$C_{14}H_{16}N_4OS_2$	$[M+H]^+$	6.68	321.0838	183.0587	5	5-100	0.9974	16.9
84	Etobenzanid	$C_{16}H_{15}Cl_2NO_3$	$[M+H]^+$	15	340.0502	121.0284	5	5-100	0.9987	35.9
85	Fenamidone	$C_{17}H_{17}N_3OS$	$[M+H]^+$	10.94	312.1165	92.0495	5	5-100	0.9984	16.3
86	Fenaminstrobin	$C_{21}H_{21}Cl_2N_3O_3$	$[M+H]^+$	16.34	434.1034	170.9763	5	5-100	0.9975	18.9
87	Fenobucarb	$C_{12}H_{17}NO_2$	$[M+H]^+$	8.91	208.1332	77.0386	25	25-500	0.9963	41.3
88	Fenothiocarb	$C_{13}H_{19}NO_2S$	$[M+H]^+$	13	254.1209	72.0444	5	5-100	0.9973	14.6
89	Fenoxasulfone	$C_{14}H_{17}Cl_2NO_4S$	$[M+H]^+$	13.95	366.0331	203.0025	40	40-1000	0.9939	12.0
90	Fenpyrazamine	$C_{17}H_{21}N_3O_2S$	$[M+H]^+$	11.91	332.1427	189.0897	5	5-100	0.996	20.8
91	fenpyroximate	$C_{24}H_{27}N_3O_4$	$[M+H]^+$	18.29	422.2074	135.0441	5	5-100	0.992	24.3
92	Fensulfothion	$C_{11}H_{17}O_4PS_2$	$[M+H]^+$	7.56	309.0379	140.029	5	5-100	0.9927	28.0
93	Fensulfothion-sulfone	$C_{11}H_{17}O_5PS_2$	$[M+H]^+$	10.24	325.0328	268.9702	5	5-100	0.9957	17.2
94	Flamprop-methyl	$C_{17}H_{15}ClFNO_3$	$[M+H]^+$	12.32	336.0797	105.0335	5	5-100	0.9861	12.2
95	Fluazifop-butyl	$C_{19}H_{20}F_3NO_4$	$[M+H]^+$	17.77	384.1417	91.0542	5	5-100	0.9977	21.2
96	Flubendiamide	$C_{23}H_{22}F_7IN_2O_4S$	$[M+H]^+$	14.68	705.0125	530.9799	5	5-100	0.9977	18.8
97	Flucycloxuron	$C_{25}H_{20}ClF_2N_3O_3$	$[M+H]^+$	17.9	484.1242	132.0444	5	5-100	0.9973	24.1
98	Flufenzin	$C_{14}H_7ClF_2N_4$	$[M+H]^+$	14.16	305.04	138.0105	25	25-500	0.9982	18.2
99	Flufiprole	$C_{16}H_{10}Cl_2F_6N_4OS$	$[M+NH_4]^+$	16.44	508.0195	404.995	10	10-200	0.983	21.2
100	Flumorph	$C_{21}H_{22}FNO_4$	$[M+H]^+$	7.11	372.1606	165.0546	5	5-100	0.9975	10.9
101	Fluometuron	$C_{10}H_{11}F_3N_2O$	$[M+H]^+$	6.33	233.0896	72.0444	5	5-100	0.996	25.2
102	Fluopyram	$C_{16}H_{11}ClF_6N_2O$	$[M+H]^+$	12.24	397.0537	173.0209	5	5-100	0.9929	18.3
103	Fluquinconazole	$C_{16}H_8Cl_2FN_5O$	$[M+H]^+$	11.62	376.0163	306.9836	5	5-100	0.9983	9.3
104	Fluridone	$C_{19}H_{14}F_3NO$	$[M+H]^+$	9.44	330.11	309.096	5	5-100	0.9956	18.6
105	Flurochloridone	$C_{12}H_{10}Cl_2F_3NO$	$[M+H]^+$	13.15	312.0164	53.0386	10	10-200	0.9992	16.7
106	fluroxypyr-meptyl	$C_{15}H_{21}Cl_2FN_2O_3$	$[M+H]^+$	18.2	367.0986	254.9723	10	10-200	0.9912	24.5
107	Flurprimidol	$C_{15}H_{15}F_3N_2O_2$	$[M+H]^+$	9.46	313.1158	269.0533	5	5-100	0.9945	12.0
108	Flurtamone	$C_{18}H_{14}F_3NO_2$	$[M+H]^+$	10.04	334.1049	247.0729	5	5-100	0.9955	12.5
109	Flusilazole	$C_{16}H_{15}F_2N_3Si$	$[M+H]^+$	12.48	316.1076	247.0749	5	5-100	0.9934	10.3
110	Flutolanil	$C_{17}H_{16}F_3NO_2$	$[M+H]^+$	13.08	324.1206	262.0663	5	5-100	0.9962	14.3
111	Flutriafol	$C_{16}H_{13}F_2N_3O$	$[M+H]^+$	6.46	302.1099	70.04	5	5-80	0.9864	21.6
112	Fluxapyroxad	$C_{18}H_{12}F_5N_3O$	$[M+H]^+$	11.7	382.0973	342.0849	5	5-100	0.9996	25.8
113	Fonofos	$C_{10}H_{15}OPS_2$	$[M+H]^+$	15.41	247.0375	80.9558	5	5-100	0.9929	41.7
114	furametpyr	$C_{17}H_{20}ClN_3O_2$	$[M+H]^+$	6.73	334.1317	290.1055	5	5-100	0.9982	15.9
115	Haloxyfop-2-ethoxyethyl	$C_{19}H_{19}ClF_3NO_5$	$[M+H]^+$	17.12	434.0977	91.0542	5	5-100	0.9985	16.0
116	Haloxyfop-methyl	$C_{16}H_{13}ClF_3NO_4$	$[M+H]^+$	16.41	376.0546	272.0085	5	5-100	0.9989	28.8
117	Hexaflumuron	$C_{16}H_8Cl_2F_6N_2O_3$	$[M+H]^+$	16.14	460.9889	141.0146	49	50-1000	0.9957	17.1
118	Hexazinone	$C_{12}H_{20}N_4O_2$	$[M+H]^+$	4.73	253.1659	71.0604	5	5-100	0.9988	10.1
119	Imazalil	$C_{14}H_{14}Cl_2N_2O$	$[M+H]^+$	5.78	297.055	69.0447	5	5-100	0.9932	13.9
120	Imazamethabenz-methyl	$C_{16}H_{20}N_2O_3$	$[M+H]^+$	4.86	289.1547	86.0964	5	5-100	0.9984	53.7

121	Indanofan	$C_{20}H_{17}ClO_3$	$[M+H]^+$	13.75	341.0941	175.0754	25	25-500	0.9937	12.7
122	Indaziflam	$C_{16}H_{20}FN_5$	$[M+H]^+$	9.21	302.1782	158.0836	5	5-100	0.9974	17.8
123	Indoxacarb	$C_{22}H_{17}ClF_3N_3O_7$	$[M+H]^+$	16.77	528.078	218.0417	5	5-100	0.9907	13.7
124	Ipfencarbazone	$C_{18}H_{14}Cl_2F_2N_4O_2$	$[M+H]^+$	14.94	427.0532	156.0255	5	5-100	0.9991	17.2
125	Iprovalicarb	$C_{18}H_{28}N_2O_3$	$[M+H]^+$	10.67	321.2173	119.0855	5	5-100	0.9961	47.0
126	Isofenphos-Methyl	$C_{14}H_{22}NO_4PS$	$[M+H]^+$	15.28	332.108	121.0284	5	5-100	0.9969	29.2
127	Isofenphos-oxon	$C_{15}H_{24}NO_5P$	$[M+H]^+$	9.83	330.1465	121.0284	5	5-100	0.9933	12.9
128	Isomethiozin	$C_{12}H_{20}N_4OS$	$[M+H]^+$	13.53	269.1431	57.0699	5	5-100	0.9965	16.3
129	Isopropalin	$C_{15}H_{23}N_3O_4$	$[M+H]^+$	18.83	310.1761	188.1308	5	5-100	0.99	21.8
130	Isoproturon	$C_{12}H_{18}N_2O$	$[M+H]^+$	6.73	207.1492	72.0444	5	5-100	0.9991	12.9
131	Isopyrazam	$C_{20}H_{23}F_2N_3O$	$[M+H]^+$	15.64	360.1895	320.1758	5	5-100	0.997	14.9
132	Isoxathion	$C_{13}H_{16}NO_4PS$	$[M+H]^+$	16.37	314.061	96.9508	5	5-100	0.9989	20.4
133	Kadethrin	$C_{23}H_{24}O_4S$	$[M+H]^+$	17.45	397.1468	128.0621	5	5-100	0.9993	17.0
134	Karbutilate	$C_{14}H_{21}N_3O_3$	$[M+H]^+$	5.61	280.1656	72.0444	5	5-100	0.9985	18.5
135	Kresoxim-methyl	$C_{18}H_{19}NO_4$	$[M+H]^+$	14.43	314.1387	116.0495	5	5-100	0.9976	20.7
136	Lactofen	$C_{19}H_{15}ClF_3NO_7$	$[M+NH_4]^+$	17.66	479.0821	343.9932	5	5-100	0.9921	15.2
137	Lenacil	$C_{13}H_{18}N_2O_2$	$[M+H]^+$	5.49	235.1441	136.0393	25	25-500	0.9969	15.9
138	Linuron	$C_9H_{10}Cl_2N_2O_2$	$[M+H]^+$	9.29	249.0192	132.9606	5	5-100	0.998	13.1
139	Malaoxon	$C_{10}H_{19}O_7PS$	$[M+H]^+$	5.77	315.0662	99.0077	5	5-100	0.996	16.1
140	Malathion	$C_{10}H_{19}O_6PS_2$	$[M+H]^+$	12.6	331.0433	99.0077	5	5-100	0.9985	15.4
141	Mecarbam	$C_{10}H_{20}NO_5PS_2$	$[M+H]^+$	13.91	330.0593	142.9385	5	5-100	0.9986	16.6
142	Mefenpyr-diethyl	$C_{16}H_{18}Cl_2N_2O_4$	$[M+H]^+$	15.62	373.0716	159.9715	5	5-100	0.9963	28.5
143	Mefluidide	$C_{11}H_{13}F_3N_2O_3S$	$[M+H]^+$	5.77	311.0672	135.0917	5	5-100	0.9955	13.8
144	Mesosulfuron-methyl	$C_{17}H_{21}N_5O_9S_2$	$[M+H]^+$	6.81	504.0853	182.0533	5	5-100	0.9956	33.6
145	Metaflumizone	$C_{24}H_{16}F_6N_4O_2$	$[M+H]^+$	17.41	507.125	178.0463	25	25-500	0.999	17.6
146	Metalaxyl	$C_{15}H_{21}NO_4$	$[M+H]^+$	6.76	280.1543	45.0335	5	5-100	0.9984	19.7
147	Methiocarb	$C_{11}H_{15}NO_2S$	$[M+H]^+$	8.96	226.0896	121.0648	5	5-100	0.998	13.1
148	Methoxyfenozide	$C_{22}H_{28}N_2O_3$	$[M+H]^+$	12.57	369.2173	91.0542	5	5-100	0.9948	20.7
149	Metobromuron	$C_9H_{11}BrN_2O_2$	$[M+H]^+$	7.12	259.0077	91.0417	5	5-100	0.9976	7.2
150	Metominostrobin-(E)	$C_{16}H_{16}N_2O_3$	$[M+H]^+$	7.97	285.1234	166.0651	5	5-100	0.9954	17.3
151	Monalide	$C_{13}H_{18}ClNO$	$[M+H]^+$	13.65	240.115	85.1012	5	5-100	0.9981	25.1
152	Myclobutanil	$C_{15}H_{17}ClN_4$	$[M+H]^+$	10.75	289.1215	70.04	5	5-100	0.9983	17.0
153	Naphthalophos	$C_{16}H_{16}NO_6P$	$[M+H]^+$	8.57	350.0796	214.0499	5	5-100	0.9953	28.0
154	Naproanilide	$C_{19}H_{17}NO_2$	$[M+H]^+$	13.69	292.1334	171.0804	5	5-100	0.9976	13.8
155	Napropamide	$C_{17}H_{21}NO_2$	$[M+H]^+$	11.72	272.1645	171.0804	5	5-100	0.9959	17.8
156	Norflurazon	$C_{12}H_9ClF_3N_3O$	$[M+H]^+$	7.15	304.0459	140.0306	5	5-100	0.9984	14.9
157	Noruron	$C_{13}H_{22}N_2O$	$[M+H]^+$	7.34	223.1811	72.0444	5	5-100	0.9964	14.1
158	Novaluron	$C_{17}H_9ClF_8N_2O_4$	$[M+H]^+$	16.65	493.0196	141.0146	50	50-1000	0.9943	16.4
159	Ofurace	$C_{14}H_{16}ClNO_3$	$[M+H]^+$	6.72	282.0892	160.1121	5	5-100	0.9991	33.4
160	Orysastrobin	$C_{18}H_{25}N_5O_5$	$[M+H]^+$	11.22	392.1941	116.0495	5	5-100	0.9961	21.2
161	Oxadixyl	$C_{14}H_{18}N_2O_4$	$[M+H]^+$	5.06	279.1339	132.0808	5	5-100	0.9962	15.4
162	Oxasulfuron	$C_{17}H_{18}N_4O_6S$	$[M+H]^+$	5.28	407.102	150.0662	5	5-100	0.9964	22.2

163	Paclobutrazol	$C_{15}H_{20}ClN_3O$	$[M+H]^+$	8.77	294.1368	70.04	5	5-100	0.9986	18.2
164	Paraoxon-Ethyl	$C_{10}H_{14}NO_6P$	$[M+H]^+$	7.14	276.0632	220.0002	5	5-100	0.9995	14.1
165	Paraoxon-Methyl	$C_8H_{10}NO_6P$	$[M+H]^+$	5.07	248.0319	109.0049	5	5-100	0.9958	22.6
166	Penconazole	$C_{13}H_{15}Cl_2N_3$	$[M+H]^+$	9.98	284.0716	70.04	5	5-100	0.9945	17.8
167	Pencycuron	$C_{19}H_{21}ClN_2O$	$[M+H]^+$	15.84	329.1415	125.0153	5	5-100	0.9962	17.8
168	Pendimethalin	$C_{13}H_{19}N_3O_4$	$[M+H]^+$	17.85	282.1448	92.0495	5	5-100	0.9933	36.5
169	Pentanochlor	$C_{13}H_{18}ClNO$	$[M+H]^+$	13.54	240.115	107.073	10	10-100	0.9806	28.3
170	Penthiopyrad	$C_{16}H_{20}F_3N_3OS$	$[M+H]^+$	14.57	360.1362	256.0351	5	5-100	0.9969	14.8
171	Pethoxamid	$C_{16}H_{22}ClNO_2$	$[M+H]^+$	12.45	296.1412	91.0542	5	5-100	0.9974	16.7
172	Phenmedipham	$C_{16}H_{16}N_2O_4$	$[M+H]^+$	9.35	301.1183	136.0393	5	5-100	0.9975	14.6
173	Phenthoate	$C_{12}H_{17}O_4PS_2$	$[M+H]^+$	15.02	321.0379	79.0542	5	5-100	0.9983	13.4
174	Phosalone	$C_{12}H_{15}ClNO_4PS_2$	$[M+H]^+$	16.04	367.9941	110.9996	5	5-100	0.9949	32.4
175	Phosmet-Oxon	$C_{11}H_{12}NO_5PS$	$[M+H]^+$	4.81	302.0252	160.0393	5	5-100	0.9966	26.4
176	Phthalic Acid, Bis-Cyclohexyl Ester	$C_{20}H_{26}O_4$	$[M+H]^+$	18.87	331.1906	149.0233	5	5-100	0.9984	16.7
177	Picaridin	$C_{12}H_{23}NO_3$	$[M+H]^+$	6.74	230.1751	130.1226	5	5-100	0.9985	13.0
178	Picoxystrobin	$C_{18}H_{16}F_3NO_4$	$[M+H]^+$	14.81	368.1104	145.0648	5	5-100	0.9967	15.4
179	Piperonyl Butoxide	$C_{19}H_{30}O_5$	$[M+NH_4]^+$	17.2	356.2423	119.0855	5	5-100	0.9975	17.8
180	Pirimicarb-Desmethyl-Formamido	$C_{11}H_{16}N_4O_3$	$[M+H]^+$	5.14	253.1295	72.0444	5	5-100	0.9982	13.5
181	Pirimiphos-Ethyl	$C_{13}H_{24}N_3O_3PS$	$[M+H]^+$	17.97	334.1349	198.1059	5	5-100	0.998	18.4
182	Pretilachlor	$C_{17}H_{26}ClNO_2$	$[M+H]^+$	16.34	312.1725	252.115	5	5-100	0.9978	16.4
183	Prodiamine	$C_{13}H_{17}F_3N_4O_4$	$[M+H]^+$	17.15	351.1278	267.0336	5	5-100	0.9925	21.1
184	Propachlor	$C_{11}H_{14}ClNO$	$[M+H]^+$	7.46	212.0837	94.0651	5	5-100	0.9959	17.7
185	Propanil	$C_9H_9Cl_2NO$	$[M+H]^+$	8.17	218.0134	127.0178	25	25-500	0.9944	21.1
186	Propaphos	$C_{13}H_{21}O_4PS$	$[M+H]^+$	13.29	305.0971	221.0032	5	5-100	0.9966	19.8
187	Propargite	$C_{19}H_{26}O_4S$	$[M+NH_4]^+$	18.36	368.1886	57.0699	5	5-100	0.9963	21.1
188	Propazine	$C_9H_{16}ClN_5$	$[M+H]^+$	8.22	230.1167	146.0228	5	5-100	0.9929	15.4
189	Propetamphos	$C_{10}H_{20}NO_4PS$	$[M+H]^+$	13.05	282.0923	109.9824	25	25-500	0.9977	19.1
190	Propisochlor	$C_{15}H_{22}ClNO_2$	$[M+H]^+$	14.39	284.1412	224.0832	5	5-100	0.999	10.7
191	Prosulfocarb	$C_{14}H_{21}NOS$	$[M+H]^+$	16.58	252.1417	91.0542	5	5-100	0.9978	11.7
192	Prothioconazole-desthio	$C_{14}H_{15}Cl_2N_3O$	$[M+H]^+$	10.55	312.0664	70.04	5	5-100	0.9821	56.9
193	Prothiofos	$C_{11}H_{15}Cl_2O_2PS_2$	$[M+H]^+$	19.08	344.9701	240.9041	25	25-500	0.9908	13.8
194	Pyracarbolid	$C_{13}H_{15}NO_2$	$[M+H]^+$	6.08	218.1176	55.0178	5	5-100	0.9978	13.6
195	Pyraclonil	$C_{15}H_{15}ClN_6$	$[M+H]^+$	8.32	315.1123	169.0509	5	5-100	0.9982	19.2
196	Pyraclostrobin	$C_{19}H_{18}ClN_3O_4$	$[M+H]^+$	15.55	388.1059	194.0812	5	5-100	0.9963	20.3
197	Pyraflufen-Ethyl	$C_{15}H_{13}Cl_2F_3N_2O_4$	$[M+H]^+$	15.06	413.0277	253.0163	5	5-100	0.9967	28.4
198	Pyrametostrobin	$C_{21}H_{23}N_3O_4$	$[M+H]^+$	12.53	382.1769	194.0812	5	5-100	0.9978	31.0
199	Pyrazolynate	$C_{19}H_{16}Cl_2N_2O_4S$	$[M+H]^+$	16.01	439.0281	91.0542	5	5-100	0.9978	15.0
200	Pyrazophos	$C_{14}H_{20}N_3O_5PS$	$[M+H]^+$	15.28	374.0934	194.056	5	5-100	0.9915	24.0
201	Pyrifenox	$C_{14}H_{12}Cl_2N_2O$	$[M+H]^+$	9.54	295.0399	93.0573	5	5-100	0.9953	12.8
202	Pyrimidifen	$C_{20}H_{28}ClN_3O_2$	$[M+H]^+$	16.3	378.1943	184.0631	5	5-100	0.9983	16.2

203	Pyrimorph	$C_{22}H_{25}ClN_2O_2$	$[M+H]^+$	13.44	385.1677	242.0367	5	5-100	0.9951	45.8
204	Quinalphos	$C_{12}H_{15}N_2O_3PS$	$[M+H]^+$	14.13	299.0614	96.9508	5	5-100	0.9974	19.0
205	Quinoclamine	$C_{10}H_6ClNO_2$	$[M+H]^+$	5.16	208.016	77.0386	5	5-100	0.9969	16.1
206	Quizalofop-Ethyl	$C_{19}H_{17}ClN_2O_4$	$[M+H]^+$	16.76	373.095	91.0542	5	5-100	0.9955	12.1
207	Rabenzazole	$C_{12}H_{12}N_4$	$[M+H]^+$	6.54	213.1135	118.0526	5	5-100	0.9981	12.4
208	Rotenone	$C_{23}H_{22}O_6$	$[M+H]^+$	13.26	395.1489	191.0703	5	5-100	0.9963	<u>54.0</u>
209	Saflufenacil	$C_{17}H_{17}ClF_4N_4O_5S$	$[M+NH_4]^+$	11.26	501.0617	348.9998	5	5-100	0.9967	10.1
210	Sebuthylazine	$C_9H_{16}ClN_5$	$[M+H]^+$	7.98	230.1167	174.0541	5	5-100	0.9945	9.7
211	Simeconazole	$C_{14}H_{20}FN_3OSi$	$[M+H]^+$	7.36	294.1432	70.04	5	5-100	0.9984	11.3
212	Spinosyn A	$C_{41}H_{65}NO_{10}$	$[M+H]^+$	14.76	732.4681	142.1226	5	5-100	0.9958	22.4
213	Spinosyn D	$C_{42}H_{67}NO_{10}$	$[M+H]^+$	14.44	746.4838	142.1226	5	5-100	0.9943	16.2
214	Spirodiclofen	$C_{21}H_{24}Cl_2O_4$	$[M+H]^+$	19.01	411.1124	71.0855	25	25-500	0.9963	37.3
215	Spirotetramat	$C_{21}H_{27}NO_5$	$[M+H]^+$	10.19	374.1962	302.1751	5	5-100	0.9928	27.3
216	Spirotetramat-mono-hydroxy	$C_{18}H_{25}NO_3$	$[M+H]^+$	4.67	304.1915	119.0855	5	5-100	0.9984	26.9
217	Sulprofos	$C_{12}H_{19}O_2PS_3$	$[M+H]^+$	18.11	323.0358	218.9698	5	5-100	0.9948	15.9
218	TCMTB	$C_9H_6N_2S_3$	$[M+H]^+$	10.73	238.9766	136.0215	5	5-100	0.9972	15.6
219	Tebuconazole	$C_{16}H_{22}ClN_3O$	$[M+H]^+$	11.84	308.1524	70.04	5	5-100	0.9977	20.1
220	Tebufenozide	$C_{22}H_{28}N_2O_2$	$[M+H]^+$	14.09	353.2224	133.0648	5	5-100	0.993	15.4
221	Tepraloxydim	$C_{17}H_{24}ClNO_4$	$[M+H]^+$	5.46	342.1467	166.0863	5	5-100	0.998	13.6
222	Terbufos-Oxon	$C_9H_{21}O_3PS_2$	$[M+H]^+$	10.63	273.0745	57.0699	10	10-200	0.9982	9.7
223	Terbufos-Oxon-Sulfone	$C_9H_{21}O_5PS_2$	$[M+H]^+$	5.25	305.0641	249.0015	5	5-100	0.9985	15.0
224	Terbufos-Sulfone	$C_9H_{21}O_4PS_3$	$[M+H]^+$	12	321.0412	275.0535	5	5-100	0.9989	6.9
225	Terbufos-Sulfoxide	$C_9H_{21}O_3PS_3$	$[M+H]^+$	8.4	305.0465	130.9385	5	5-100	0.9968	17.6
226	Terbumeton	$C_{10}H_{19}N_5O$	$[M+H]^+$	5.61	226.1662	170.1036	5	5-100	0.9982	15.9
227	Terbutryn	$C_{10}H_{19}N_5S$	$[M+H]^+$	9.53	242.1434	186.0808	5	5-100	0.9941	9.1
228	Tetrachlorvinphos	$C_{10}H_9Cl_4O_4P$	$[M+H]^+$	12.81	364.9065	127.0155	5	5-100	0.9976	9.6
229	Tiadinil	$C_{11}H_{10}ClN_3OS$	$[M+H]^+$	10.27	268.0306	101.0168	5	5-100	0.9921	17.0
230	Tolfenpyrad	$C_{21}H_{22}ClN_3O_2$	$[M+H]^+$	17.04	384.1477	197.0961	5	5-100	0.9912	17.5
231	Triadimefon	$C_{14}H_{16}ClN_3O_2$	$[M+H]^+$	11.26	294.1004	57.0699	5	5-100	0.9983	20.6
232	Triadimenol	$C_{14}H_{18}ClN_3O_2$	$[M+H]^+$	8.58	296.1158	70.04	5	5-100	0.999	8.4
233	Triamiphos	$C_{12}H_{19}N_6OP$	$[M+H]^+$	5.54	295.1431	135.0679	5	5-100	0.9985	16.0
234	Triapenthenol	$C_{15}H_{25}N_3O$	$[M+H]^+$	11.45	264.207	70.04	5	5-100	0.9989	31.8
235	Triazophos	$C_{12}H_{16}N_3O_3PS$	$[M+H]^+$	12.9	314.0723	119.0604	5	5-100	0.9965	38.0
236	Tribufos	$C_{12}H_{27}OPS_3$	$[M+H]^+$	18.98	315.1029	168.9905	5	5-100	0.9987	19.0
237	Tributyl Phosphate	$C_{12}H_{27}O_4P$	$[M+H]^+$	14.85	267.171	98.9842	5	5-100	0.9989	21.0
238	Tridemorph	$C_{19}H_{39}NO$	$[M+H]^+$	13.82	298.3104	57.0699	50	50-1000	0.9986	11.8
239	Trifloxystrobin	$C_{20}H_{19}F_3N_2O_4$	$[M+H]^+$	16.78	409.137	145.026	5	5-100	0.997	20.6
240	Triticonazole	$C_{17}H_{20}ClN_3O$	$[M+H]^+$	6.95	318.1368	70.04	5	5-100	0.9988	26.5
241	Tritosulfuron	$C_{13}H_9F_6N_5O_4S$	$[M+H]^+$	9.4	446.0352	145.0258	5	5-100	0.9945	10.7
242	Valifenalate	$C_{19}H_{27}ClN_2O_5$	$[M+H]^+$	10.31	399.1681	116.0706	5	5-100	0.996	15.4
243	Zoxamide	$C_{14}H_{16}Cl_3NO_2$	$[M+H]^+$	15.09	336.0319	186.9712	5	5-100	0.9953	18.9
244	1,3-Diphenyl Urea	$C_{13}H_{12}N_2O$	$[M+H]^+$	7.08	213.1022	77.0386	5	5-100	0.9987	18.7

245	2,6-Dichlorobenzamide	$C_7H_5Cl_2NO$	$[M+H]^+$	3.16	189.9821	172.9556	50	50-1000	0.9983	34.8
246	Abamectin	$C_{48}H_{72}O_{14}$	$[M+NH_4]^+$	18.75	890.526	567.3319	5	5-100	0.9972	14.0
247	Acephate	$C_4H_{10}NO_3PS$	$[M+H]^+$	0.82	184.0192	142.9926	5	5-100	0.9968	19.1
248	Acetochlor	$C_{14}H_{20}ClNO_2$	$[M+H]^+$	12.62	270.1255	133.0886	5	5-100	0.9981	13.5
249	Aclonifen	$C_{12}H_9ClN_2O_3$	$[M+H]^+$	13.81	265.0373	248.0347	50	50-1000	0.9934	27.3
250	Amisulbrom	$C_{13}H_{13}BrFN_5O_4S_2$	$[M+H]^+$	16.79	465.9649	226.973	50	50-1000	0.9971	23.3
251	Anilofos	$C_{13}H_{19}ClNO_3PS_2$	$[M+H]^+$	14.79	368.0299	124.9821	5	5-100	0.9958	68.1
252	Aziprotryne	$C_7H_{11}N_7S$	$[M+H]^+$	9.65	226.0869	68.0243	5	5-100	0.9928	26.1
253	Azoxystrobin	$C_{22}H_{17}N_3O_5$	$[M+H]^+$	11.17	404.1241	329.0795	5	5-100	0.9932	13.7
254	Benalaxyl	$C_{20}H_{23}NO_3$	$[M+H]^+$	14.11	326.1751	91.0542	5	5-100	0.9945	16.4
255	Benodanil	$C_{13}H_{10}INO$	$[M+H]^+$	8.39	323.988	230.9301	5	5-100	0.9959	14.4
256	Benzobicyclon	$C_{22}H_{19}ClO_4S_2$	$[M+H]^+$	13.07	447.0488	257.0631	5	5-100	0.9972	16.1
257	Benzofenap	$C_{22}H_{20}Cl_2N_2O3$	$[M+H]^+$	16.23	431.0931	105.0699	5	5-100	0.9979	13.1
258	Benzovindiflupyr	$C_{18}H_{15}Cl_2F2N3O$	$[M+H]^+$	14.43	398.064	159.0364	5	5-100	0.9899	32.1
259	Bitertanol	$C_{20}H_{23}N_3O_2$	$[M+H]^+$	12.77	338.1863	70.04	5	5-100	0.9866	12.7
260	Boscalid	$C_{18}H_{12}Cl_2N_2O$	$[M+H]^+$	11.3	343.0399	271.0866	5	5-100	0.9984	34.1
261	Bromacil	$C_9H_{13}BrN_2O_2$	$[M+H]^+$	4.93	261.0233	131.9443	10	10-200	0.9947	55.2
262	Bromobutide	$C_{15}H_{22}BrNO$	$[M+H]^+$	13.8	312.0958	119.0855	5	5-100	0.9979	15.9
263	Bromophos-Ethyl	$C_{10}H_{12}BrCl_2O_3PS$	$[M+H]^+$	18.8	392.8878	161.9632	50	50-1000	0.9929	30.9
264	Brompyrazon	$C_{10}H_8BrN_3O$	$[M+H]^+$	3.81	265.9924	92.0495	5	5-100	0.9979	16.0
265	Bromuconazole	$C_{13}H_{12}BrCl_2N_3O$	$[M+H]^+$	10.46	375.9614	158.9763	5	5-100	0.9967	9.6
266	Butafenacil	$C_{20}H_{18}ClF_3N_2O_6$	$[M+NH_4]^+$	14.21	492.1157	331.0092	5	5-100	0.9937	8.7
267	Butamifos	$C_{13}H_{21}N_2O_4PS$	$[M+H]^+$	16.5	333.1035	95.9668	5	5-100	0.9905	10.4
268	Butoxycarboxim	$C_7H_{14}N_2O_4S$	$[M+H]^+$	2.66	223.0747	44.0495	5	5-100	0.99	12.6
269	Cadusafos	$C_{10}H_{23}O_2PS_2$	$[M+H]^+$	14.78	271.095	96.9508	5	5-100	0.9971	13.5
270	Cafenstrole	$C_{16}H_{22}N_4O_3S$	$[M+H]^+$	12.85	351.1485	72.0444	5	5-100	0.9855	14.8
271	Carbaryl	$C_{12}H_{11}NO_2$	$[M+H]^+$	6.29	202.0863	127.0542	5	5-100	0.9968	12.9
272	Carbetamide	$C_{12}H_{16}N_2O_3$	$[M+H]^+$	4.66	237.1234	72.0808	5	5-100	0.9984	19.5
273	Carboxin	$C_{12}H_{13}NO_2S$	$[M+H]^+$	6.54	236.074	93.0573	5	5-100	0.999	18.6
274	Chlorbromuron	$C_9H_{10}BrClN_2O_2$	$[M+H]^+$	9.88	292.9687	203.9205	10	10-200	0.9974	17.5
275	Chlorfluazuron	$C_{20}H_9Cl_3F_5N_3O_3$	$[M+H]^+$	18.19	539.9702	382.9358	25	25-500	0.9977	41.1
276	Chloroxuron	$C_{15}H_{15}ClN_2O_2$	$[M+H]^+$	10.16	291.0895	72.0449	5	5-100	0.9962	23.0
277	Chlorthiophos	$C_{11}H_{15}Cl_2O_3PS_2$	$[M+H]^+$	18.17	360.965	191.956	50	50-1000	0.9984	53.3
278	Clodinafop-propargyl	$C_{17}H_{13}ClFNO_4$	$[M+H]^+$	15.12	350.059	91.0542	5	5-100	0.9994	16.6
279	Clofentezine	$C_{14}H_8Cl_2N_4$	$[M+H]^+$	15.4	303.0199	102.0338	5	5-100	0.9923	16.9
280	Clomazone	$C_{12}H_{14}ClNO_2$	$[M+H]^+$	8	240.0786	125.0153	5	5-100	0.9993	17.9
281	Clomeprop	$C_{16}H_{15}Cl_2NO_2$	$[M+H]^+$	16.6	324.0562	120.0808	5	5-100	0.9994	24.8
282	Coumaphos	$C_{14}H_{16}ClO_5PS$	$[M+H]^+$	15.61	363.0217	226.9926	5	5-100	0.9981	26.4
283	Crotoxyphos	$C_{14}H_{19}O_6P$	$[M+NH_4]^+$	9.67	332.1258	211.0366	5	5-100	0.9961	25.6
284	Cyanazine	$C_9H_{13}ClN_6$	$[M+H]^+$	5.22	241.0963	214.0854	5	5-100	0.9925	23.3
285	Cyanofenphos	$C_{15}H_{14}NO_2PS$	$[M+H]^+$	15.62	304.0556	156.9872	50	50-1000	0.9939	25.4
286	Cyazofamid	$C_{13}H_{13}ClN_4O_2S$	$[M+H]^+$	14.26	325.0521	108.0114	5	5-100	0.9944	21.1

287	Cycluron	$C_{11}H_{22}N_2O$	$[M+H]^+$	6.49	199.1805	72.0444	5	5-100	0.9975	22.8
288	Cyenopyrafen	$C_{24}H_{31}N_3O_2$	$[M+H]^+$	18.65	394.2489	310.1914	5	5-100	0.9964	14.5
289	Cyflumetofen	$C_{24}H_{24}F_3NO_4$	$[M+NH_4]^+$	17.8	465.1996	173.0209	5	5-100	0.9871	17.9
290	Cymoxanil	$C_7H_{10}N_4O_3$	$[M+H]^+$	2.61	199.0826	53.0134	50	50-1000	0.997	31.1
291	Diallate	$C_{10}H_{17}Cl_2NOS$	$[M+H]^+$	16.72	270.0481	86.06	25	25-500	0.9965	35.3
292	Dichlorvos	$C_4H_7Cl_2O_4P$	$[M+H]^+$	5.24	220.9532	109.0049	50	50-1000	0.9954	20.1
293	Diethofencarb	$C_{14}H_{21}NO_4$	$[M+H]^+$	9.58	268.1543	124.0393	5	5-100	0.9909	13.4
294	Diethyltoluamide	$C_{12}H_{17}NO$	$[M+H]^+$	6.72	192.1383	91.0542	5	5-100	0.9959	15.8
295	Diflufenican	$C_{19}H_{11}F_5N_2O_2$	$[M+H]^+$	16.41	395.0814	266.0412	5	5-100	0.999	16.9
296	Dimefuron	$C_{15}H_{19}ClN_4O_3$	$[M+H]^+$	8.12	339.1218	72.0444	5	5-100	0.9852	24.3
297	Dimethomorph	$C_{21}H_{22}ClNO_4$	$[M+H]^+$	8.46	388.131	165.0546	5	5-100	0.9927	26.7
298	Dimethylvinphos (E)	$C_{10}H_{10}Cl_3O_4P$	$[M+H]^+$	11.58	330.9455	127.0155	5	5-100	0.9956	10.5
299	Dimethylvinphos (Z)	$C_{10}H_{10}Cl_3O_4P$	$[M+H]^+$	10.59	330.9455	127.0155	5	5-100	0.9957	19.8
300	Dimetilan	$C_{10}H_{16}N_4O_3$	$[M+H]^+$	3.86	241.1295	72.0444	5	5-100	0.9969	32.3
301	Dimoxystrobin	$C_{19}H_{22}N_2O_3$	$[M+H]^+$	13.19	327.1703	116.0495	5	5-100	0.9927	25.0
302	Diniconazole	$C_{15}H_{17}Cl_2N_3O$	$[M+H]^+$	13.05	326.0821	70.04	5	5-100	0.9976	22.1
303	Dinitramine	$C_{11}H_{13}F_3N_4O_4$	$[M+H]^+$	15.02	323.0962	305.0856	25	25-500	0.9974	15.1
304	Dipropyl Isocinchomeronate	$C_{13}H_{17}NO_4$	$[M+H]^+$	10.32	252.124	164.0706	5	5-100	0.9956	10.4
305	Disulfoton sulfoxide	$C_8H_{19}O_3PS_3$	$[M+H]^+$	6.38	291.0304	213.0167	5	5-100	0.9931	15.8
306	Diuron	$C_9H_{10}Cl_2N_2O$	$[M+H]^+$	6.72	233.0243	72.0449	5	5-100	0.9972	17.9
307	DMST	$C_9H_{14}N_2O_2S$	$[M+H]^+$	6.38	215.0849	77.0386	50	50-1000	0.997	18.9
308	Edifenphos	$C_{14}H_{15}O_2PS_2$	$[M+H]^+$	13.54	311.0324	109.0107	5	5-100	0.9967	19.4
309	EPN	$C_{14}H_{14}NO_4PS$	$[M+H]^+$	16.59	324.0454	296.0141	25	25-500	0.9994	20.5
310	Epoxiconazole	$C_{17}H_{13}ClFN_3O$	$[M+H]^+$	11.27	330.0806	121.0448	5	5-100	0.9959	23.4
311	EPTC	$C_9H_{19}NOS$	$[M+H]^+$	13.01	190.126	128.107	50	50-1000	0.9853	31.2
312	Esprocarb	$C_{15}H_{23}NOS$	$[M+H]^+$	17.21	266.1575	91.0542	5	5-100	0.999	13.2
313	Ethiofencarb	$C_{11}H_{15}NO_2S$	$[M+H]^+$	6.61	226.0896	107.0491	5	5-100	0.9885	19.1
314	Ethiofencarb-sulfone	$C_{11}H_{15}NO_4S$	$[M+NH_4]^+$	3.6	258.0795	107.0491	5	5-100	0.9968	17.7
315	Ethiofencarb-sulfoxide	$C_{11}H_{15}NO_3S$	$[M+H]^+$	3.21	242.0845	107.0491	5	5-100	0.9948	24.1
316	Ethion	$C_9H_{22}O_4P_2S_4$	$[M+H]^+$	17.97	384.9949	199.0011	5	5-100	0.9928	9.3
317	Ethoprophos	$C_8H_{19}O_2PS_2$	$[M+H]^+$	10.96	243.0637	96.9508	5	5-100	0.9961	8.7
318	ethychlozate	$C_{11}H_{11}ClN_2O_2$	$[M+H]^+$	7.08	239.0583	165.0214	5	5-100	0.9913	21.3
319	Etrimfos	$C_{10}H_{17}N_2O_4PS$	$[M+H]^+$	14.61	293.0719	124.9821	5	5-100	0.9945	22.4
320	Famphur	$C_{10}H_{16}NO_5PS_2$	$[M+H]^+$	9.45	326.028	93.01	5	5-100	0.9982	15.9
321	Fenamiphos	$C_{13}H_{22}NO_3PS$	$[M+H]^+$	10.6	304.1131	201.9848	5	5-100	0.9924	16.3
322	Fenamiphos-sulfone	$C_{13}H_{22}NO_5PS$	$[M+H]^+$	5.65	336.1029	266.0247	5	5-100	0.9924	18.5
323	Fenamiphos-sulfoxide	$C_{13}H_{22}NO_4PS$	$[M+H]^+$	4.65	320.108	108.0573	5	5-100	0.9934	21.0

324	Fenarimol	$C_{17}H_{12}Cl_2N_2O$	$[M+H]^+$	10.69	331.0399	81.0447	5	5-100	0.9971	19.4
325	Fenazaquin	$C_{20}H_{22}N_2O$	$[M+H]^+$	18.44	307.1805	57.0699	5	5-100	0.9992	10.8
326	Fenbuconazole	$C_{19}H_{17}ClN_4$	$[M+H]^+$	12.5	337.1215	70.04	5	5-100	0.9967	14.3
327	Fenchlorphos-Oxon	$C_8H_8Cl_3O_4P$	$[M+H]^+$	10.42	304.9298	109.0049	50	50-1000	0.9967	22.2
328	Fenfuram	$C_{12}H_{11}NO_2$	$[M+H]^+$	6.76	202.0863	109.0284	5	5-100	0.9946	13.1
329	Fenhexamid	$C_{14}H_{17}Cl_2NO_2$	$[M+H]^+$	11.15	302.0709	55.0542	25	25-500	0.9986	31.0
330	Fenoxanil	$C_{15}H_{18}Cl_2N_2O_2$	$[M+H]^+$	14.11	329.0818	86.0964	5	5-100	0.9973	12.4
331	Fenoxaprop-Ethyl	$C_{18}H_{16}ClNO_5$	$[M+H]^+$	16.66	362.079	91.0542	50	50-1000	0.9977	44.7
332	Fenoxycarb	$C_{17}H_{19}NO_4$	$[M+H]^+$	13.04	302.1387	88.0393	5	5-100	0.9986	15.4
333	Fensulfothion-Oxon-Sulfone	$C_{11}H_{17}O_6PS$	$[M+H]^+$	4.86	309.0565	252.993	5	5-100	0.9971	16.9
334	Fenthion	$C_{10}H_{15}O_3PS_2$	$[M+H]^+$	8.91	279.0273	185.9899	50	50-1000	0.996	11.9
335	Fenthion-oxon	$C_{10}H_{15}O_4PS$	$[M+H]^+$	7.27	263.0501	44.9794	5	5-100	0.9964	20.7
336	Fenthion-oxon-sulfone	$C_{10}H_{15}O_6PS$	$[M+H]^+$	4.12	295.04	91.0542	5	5-100	0.998	18.2
337	Fenthion-sulfone	$C_{10}H_{15}O_5PS_2$	$[M+H]^+$	7.77	311.0171	124.9821	25	25-500	0.9949	24.9
338	Fenthion-sulfoxide	$C_{10}H_{15}O_4PS_2$	$[M+H]^+$	6.06	295.0222	109.0049	5	5-100	0.9964	12.5
339	Ferimzone	$C_{15}H_{18}N_4$	$[M+H]^+$	5.63	255.1612	132.0808	5	5-100	0.9987	22.5
340	Flamprop-isopropyl	$C_{19}H_{19}ClFNO_3$	$[M+H]^+$	15.14	364.111	105.0335	5	5-100	0.9971	21.5
341	Fluacrypyrim	$C_{20}H_{21}F_3N_2O_5$	$[M+H]^+$	16.71	427.1475	145.0648	5	5-100	0.9976	28.7
342	Fluazuron	$C_{20}H_{10}Cl_2F_5N_3O_3$	$[M+H]^+$	17.43	506.0092	141.0135	5	5-100	0.9962	20.5
343	Flufenacet	$C_{14}H_{13}F_4N_3O_2S$	$[M+H]^+$	13.11	364.0737	124.0557	5	5-100	0.994	15.2
344	Flufenoxuron	$C_{21}H_{11}ClF_6N_2O_3$	$[M+H]^+$	17.81	489.0435	141.0147	50	50-1000	0.9957	31.5
345	Flufenpyr-Ethyl	$C_{16}H_{13}ClF_4N_2O_4$	$[M+NH_4]^+$	13.92	409.0573	307.0256	5	5-100	0.9897	25.7
346	Flumetralin	$C_{16}H_{12}ClF_4N_3O_4$	$[M+H]^+$	17.98	422.0525	143.0087	49	50-1000	0.9915	18.4
347	Flumiclorac-Pentyl	$C_{21}H_{23}ClFNO_5$	$[M+H]^+$	17.51	441.1593	308.0484	5	5-100	0.9958	12.6
348	Flumioxazin	$C_{19}H_{15}FN_2O_4$	$[M+H]^+$	9.87	355.1088	148.0557	50	50-1000	0.9896	27.8
349	Fluopicolide	$C_{14}H_8Cl_3F_3N_2O$	$[M+H]^+$	11.97	382.9727	172.9556	5	5-100	0.9994	21.6
350	Fluoxastrobin	$C_{21}H_{16}ClFN_4O_5$	$[M+H]^+$	13.54	459.0866	188.038	5	5-100	0.9932	27.8
351	Fluthiacet-methyl	$C_{15}H_{15}ClFN_3O_3S_2$	$[M+H]^+$	13.86	404.03	214.9831	5	5-100	0.9876	16.9
352	Fosthiazate	$C_9H_{18}NO_3PS_2$	$[M+H]^+$	6.44	284.0539	104.0165	5	5-100	0.9969	9.5
353	Furalaxyl	$C_{17}H_{19}NO_4$	$[M+H]^+$	9.38	302.1387	95.0128	5	5-100	0.9888	20.9
354	Furathiocarb	$C_{18}H_{26}N_2O_5S$	$[M+H]^+$	17.31	383.1635	195.0474	5	5-100	0.9982	21.5
355	Griseofulvin	$C_{17}H_{17}ClO_6$	$[M+H]^+$	7.09	353.0786	69.0335	5	5-100	0.9981	22.5
356	Halofenozide	$C_{18}H_{19}ClN_2O_2$	$[M+H]^+$	10.31	331.1208	105.0335	50	50-1000	0.9982	25.1
357	Heptenophos	$C_9H_{12}ClO_4P$	$[M+H]^+$	7.15	251.0235	109.0049	5	5-100	0.9984	13.2
358	Hexaconazole	$C_{14}H_{17}Cl_2N_3O$	$[M+H]^+$	12.29	314.0825	70.04	5	5-100	0.9964	29.5
359	Hexythiazox	$C_{17}H_{21}ClN_2O_2S$	$[M+H]^+$	17.76	353.1085	168.057	5	5-100	0.9916	21.0
360	Imibenconazole	$C_{17}H_{13}Cl_3N_4S$	$[M+H]^+$	16.49	410.9999	125.0153	5	5-100	0.9971	10.7
361	Imidacloprid-Urea	$C_9H_{10}ClN_3O$	$[M+H]^+$	3.3	212.0585	100.1121	5	5-100	0.9978	16.9
362	Imidaclothiz	$C_7H_8ClN_5O_2S$	$[M+H]^+$	3.86	262.016	122.0713	10	10-200	0.9937	26.6
363	Imiprothrin	$C_{17}H_{22}N_2O_4$	$[M+H]^+$	12.49	319.1657	151.1117	25	25-500	0.9875	15.2
364	Ipconazole	$C_{18}H_{24}ClN_3O$	$[M+H]^+$	14.23	334.1681	70.04	5	5-100	0.9929	17.9
365	Iprobenfos	$C_{13}H_{21}O_3PS$	$[M+H]^+$	12.4	289.1022	91.0542	5	5-100	0.9951	22.8

366	Isazofos	$C_9H_{17}ClN_3O_3PS$	$[M+H]^+$	13.69	314.049	119.9957	5	5-100	0.9956	26.9
367	Isoprocarb	$C_{11}H_{15}NO_2$	$[M+H]^+$	7.11	194.1176	95.0491	10	10-200	0.9939	24.1
368	Isoprothiolane	$C_{12}H_{18}O_4S_2$	$[M+H]^+$	12.29	291.0719	144.9776	5	5-100	0.9969	28.9
369	Isouron	$C_{10}H_{17}N_3O_2$	$[M+H]^+$	5.08	212.1387	72.0444	5	5-100	0.9987	16.0
370	Isoxaben	$C_{18}H_{24}N_2O_4$	$[M+H]^+$	12.13	333.1809	165.0546	5	5-100	0.9944	24.0
371	Isoxadifen-ethyl	$C_{18}H_{17}NO_3$	$[M+H]^+$	14.48	296.1281	232.0757	5	5-100	0.999	19.4
372	Isoxaflutole	$C_{15}H_{12}F_3NO_4S$	$[M+H]^+$	4.47	360.0512	250.9984	50	50-1000	0.9993	28.0
373	Mandipropamid	$C_{23}H_{22}ClNO_4$	$[M+H]^+$	11.95	412.131	125.0153	5	5-100	0.9887	21.5
374	Mefenacet	$C_{16}H_{14}N_2O_2S$	$[M+H]^+$	10.95	299.0849	120.0808	5	5-100	0.9957	25.2
375	Mepanipyrim	$C_{14}H_{13}N_3$	$[M+H]^+$	11.59	224.1182	77.0386	5	5-100	0.9926	21.7
376	Mephosfolan	$C_8H_{16}NO_3PS_2$	$[M+H]^+$	4.93	270.0382	139.9566	5	5-100	0.9982	24.2
377	Mepronil	$C_{17}H_{19}NO_2$	$[M+H]^+$	12.27	270.1489	91.0542	5	5-100	0.9967	19.6
378	Metamifop	$C_{23}H_{18}ClFN_2O_4$	$[M+H]^+$	16.5	441.1025	288.0422	50	50-1000	0.9944	12.3
379	Methabenzthiazuron	$C_{10}H_{11}N_3OS$	$[M+H]^+$	6.01	222.0696	165.0481	5	5-100	0.995	13.9
380	Methidathion	$C_6H_{11}N_2O_4PS_3$	$[M+H]^+$	9.2	302.9691	58.0287	10	10-200	0.9975	12.7
381	Methoprotryne	$C_{11}H_{21}N_5OS$	$[M+H]^+$	6.56	272.154	170.0495	5	5-100	0.9989	29.4
382	Metolachlor	$C_{15}H_{22}ClNO_2$	$[M+H]^+$	12.41	284.1412	252.115	5	5-100	0.993	11.7
383	Metolcarb	$C_9H_{11}NO_2$	$[M+H]^+$	5.15	166.0863	94.0413	25	25-500	0.9993	27.5
384	Metominostrobin-(Z)	$C_{16}H_{16}N_2O_3$	$[M+H]^+$	7.17	285.1234	77.0386	5	5-100	0.9968	20.7
385	Metoxuron	$C_{10}H_{13}ClN_2O_2$	$[M+H]^+$	4.63	229.0738	72.0444	5	5-100	0.9933	9.8
386	Metrafenone	$C_{19}H_{21}BrO_5$	$[M+H]^+$	16.32	409.0645	209.0808	5	5-100	0.9968	13.1
387	Metribuzin	$C_8H_{14}N_4OS$	$[M+H]^+$	5.33	215.0961	49.0106	5	5-100	0.9949	12.3
388	Molinate	$C_9H_{17}NOS$	$[M+H]^+$	10.09	188.1104	55.0542	50	50-1000	0.9921	24.6
389	Monolinuron	$C_9H_{11}ClN_2O_2$	$[M+H]^+$	6.65	215.0582	98.9996	5	5-100	0.9957	16.4
390	Monuron	$C_9H_{11}ClN_2O$	$[M+H]^+$	5.01	199.0633	72.0444	5	5-100	0.9946	26.5
391	Neburon	$C_{12}H_{16}Cl_2N_2O$	$[M+H]^+$	13.22	275.0713	57.0699	5	5-100	0.998	26.0
392	Nitralin	$C_{13}H_{19}N_3O_6S$	$[M+H]^+$	14.34	346.1067	304.0598	25	25-500	0.9924	26.5
393	Orbencarb	$C_{12}H_{16}ClNOS$	$[M+H]^+$	14.9	258.0714	125.0153	5	5-100	0.9978	19.6
394	Oryzalin	$C_{12}H_{18}N_4O_6S$	$[M+H]^+$	12.97	347.102	198.0134	50	50-1000	0.9913	17.1
395	Oxadiargyl	$C_{15}H_{14}Cl_2N_2O_3$	$[M+H]^+$	15.78	341.0454	223.0031	50	50-1000	0.9953	30.3
396	Oxaziclomefone	$C_{20}H_{19}Cl_2NO_2$	$[M+H]^+$	17.37	376.0866	161.0597	5	5-100	0.9976	14.5
397	Oxycarboxin	$C_{12}H_{13}NO_4S$	$[M+H]^+$	4.46	268.0638	175.006	5	5-100	0.9974	28.9
398	Pebulate	$C_{10}H_{21}NOS$	$[M+H]^+$	15.37	204.1417	57.0699	50	50-1000	0.9926	17.2
399	Penflufen	$C_{18}H_{24}FN_3O$	$[M+H]^+$	13.04	318.1989	234.1037	5	5-100	0.9987	9.9
400	Phorate	$C_7H_{17}O_2PS_3$	$[M+H]^+$	15.79	261.0201	46.995	50	50-1000	0.9946	27.6
401	Phorate-oxon	$C_7H_{17}O_3PS_2$	$[M+H]^+$	7.1	245.0428	75.0263	5	5-100	0.9966	11.7
402	Phorate-oxon-sulfone	$C_7H_{17}O_5PS_2$	$[M+H]^+$	4.2	277.0337	110.9664	5	5-100	0.9969	20.6
403	Phorate-oxon-sulfoxide	$C_7H_{17}O_4PS_2$	$[M+H]^+$	3.5	261.0385	110.9664	5	5-100	0.9953	24.6
404	Phorate-Sulfone	$C_7H_{17}O_4PS_3$	$[M+H]^+$	8.65	293.0097	96.9508	5	5-100	0.9986	14.9
405	Phorate-Sulfoxide	$C_7H_{17}O_3PS_3$	$[M+H]^+$	6.37	277.015	96.9508	5	5-100	0.9975	15.2
406	Phosmet	$C_{11}H_{12}NO_4PS_2$	$[M+H]^+$	10.34	318.0018	160.0393	10	10-200	0.9986	16.5

407	Phosphamidon	$C_{10}H_{19}ClNO_5P$	$[M+H]^+$	4.73	300.0762	127.0155	5	5-100	0.9973	28.6
408	Phoxim	$C_{12}H_{15}N_2O_3PS$	$[M+H]^+$	16.05	299.0614	77.0389	25	25-500	0.9953	18.1
409	Phthalic Acid, Benzyl Butyl Ester	$C_{19}H_{20}O_4$	$[M+H]^+$	16.68	313.1437	149.0233	5	5-100	0.9993	9.5
410	Phthalic Acid, Bis-Butyl Ester	$C_{16}H_{22}O_4$	$[M+H]^+$	16.89	279.1586	149.0233	25	25-500	0.9903	19.6
411	Picolinafen	$C_{19}H_{12}F_4N_2O_2$	$[M+H]^+$	17.09	377.0908	238.0474	5	5-100	0.999	16.6
412	Pinoxaden	$C_{23}H_{32}N_2O_4$	$[M+H]^+$	13.25	401.2435	57.0699	5	5-100	0.9971	14.8
413	Piperophos	$C_{14}H_{28}NO_3PS_2$	$[M+H]^+$	16.25	354.1321	170.9334	5	5-100	0.9977	20.9
414	Pirimiphos-Methyl	$C_{11}H_{20}N_3O_3PS$	$[M+H]^+$	15.91	306.1036	164.1182	5	5-100	0.9974	22.1
415	Pirimiphos-methyl-N-desethyl	$C_9H_{16}N_3O_3PS$	$[M+H]^+$	7.5	278.0723	67.0291	5	5-100	0.998	24.7
416	Prochloraz	$C_{15}H_{16}Cl_3N_3O_2$	$[M+H]^+$	13.12	376.0381	70.0287	5	5-100	0.9977	23.6
417	Profenofos	$C_{11}H_{15}BrClO_3PS$	$[M+H]^+$	16.19	372.9424	96.9509	5	5-100	0.9987	21.4
418	Promecarb	$C_{12}H_{17}NO_2$	$[M+H]^+$	9.8	208.1332	91.0542	5	5-100	0.9936	17.1
419	Prometon	$C_{10}H_{19}N_5O$	$[M+H]^+$	5.3	226.1662	142.0723	5	5-100	0.9983	24.1
420	Prometryn	$C_{10}H_{19}N_5S$	$[M+H]^+$	8.68	242.1434	68.0243	5	5-100	0.9965	15.3
421	Propaquizafop	$C_{22}H_{22}ClN_3O_5$	$[M+H]^+$	16.96	444.1321	56.0495	5	5-100	0.9915	19.1
422	Propiconazole	$C_{15}H_{17}Cl_2N_3O_2$	$[M+H]^+$	13.16	342.0771	69.0699	5	5-100	0.9953	25.8
423	Propoxur	$C_{11}H_{15}NO_3$	$[M+H]^+$	5.73	210.1125	111.0441	5	5-100	0.9969	17.5
424	Propyzamide	$C_{12}H_{11}Cl_2NO$	$[M+H]^+$	11.12	256.0291	189.9821	5	5-100	0.9938	15.1
425	Proquinazid	$C_{14}H_{17}IN_2O_2$	$[M+H]^+$	18.29	373.0408	330.9938	5	5-100	0.9984	13.6
426	Prothoate	$C_9H20NO_3PS_2$	$[M+H]^+$	7.87	286.0695	96.9508	5	5-100	0.9966	16.1
427	Pyraclofos	$C_{14}H_{18}ClN_2O_3PS$	$[M+H]^+$	14.72	361.0537	138.0103	5	5-100	0.9958	19.7
428	Pyraoxystrobin	$C_{22}H_{21}ClN_2O_4$	$[M+H]^+$	14.95	413.1272	145.0648	5	5-100	0.9896	12.1
429	Pyrazoxyfen	$C_{20}H_{16}Cl_2N_2O_3$	$[M+H]^+$	13.95	403.0612	105.0335	5	5-100	0.9964	18.8
430	Pyrethrins	$C_{21}H_{28}O_3$	$[M+H]^+$	18.4	329.2111	105.0699	50	50-1000	0.9894	16.8
431	Pyribenzoxim	$C_{32}H_{27}N_5O_8$	$[M+H]^+$	17.8	610.1923	180.0808	25	25-500	0.9899	24.5
432	Pyributicarb	$C_{18}H_{22}N_2O_2S$	$[M+H]^+$	17.85	331.1475	181.043	5	5-100	0.9979	10.8
433	Pyridaben	$C_{19}H_{25}ClN_2OS$	$[M+H]^+$	18.85	365.1449	147.1168	5	5-100	0.9947	28.3
434	Pyridaphenthion	$C_{14}H_{17}N_2O_4PS$	$[M+H]^+$	11.69	341.0719	92.0498	5	5-100	0.9967	14.3
435	Pyriftalid	$C_{15}H_{14}N_2O_4S$	$[M+H]^+$	10.63	319.0747	139.0502	55	55-1100	0.996	58.6
436	Pyrimethanil	$C_{12}H_{13}N_3$	$[M+H]^+$	7.56	200.1182	77.0386	5	5-100	0.9968	14.3
437	Pyriminobac-Methyl (Z)	$C_{17}H_{19}N_3O_6$	$[M+H]^+$	9.3	362.1347	174.055	5	5-100	0.9961	14.3
438	Pyrimitate	$C_{11}H_{20}N_3O_3PS$	$[M+H]^+$	14.73	306.1036	154.0975	5	5-100	0.9962	16.2
439	Pyriproxyfen	$C_{20}H_{19}NO_3$	$[M+H]^+$	17.56	322.1438	96.0444	5	5-100	0.9981	24.4
440	Pyrisoxazole	$C_{16}H_{17}ClN_2O$	$[M+H]^+$	6.58	289.1105	120.0444	5	5-100	0.9917	19.3
441	Pyroquilon	$C_{11}H_{11}NO$	$[M+H]^+$	4.91	174.0913	117.0573	5	5-100	0.9992	12.4
442	Quinoxyfen	$C_{15}H_8Cl_2FNO$	$[M+H]^+$	16.82	308.004	196.9789	5	5-100	0.9986	12.9
443	Sebuthylazine-desethyl	$C_7H_{12}ClN_5$	$[M+H]^+$	4.59	202.0854	146.0228	5	5-100	0.9911	19.1
444	Secbumeton	$C_{10}H_{19}N_5O$	$[M+H]^+$	5.24	226.1662	170.1036	5	5-100	0.9983	13.8
445	Sedaxane	$C_{18}H_{19}F_2N_3O$	$[M+H]^+$	11.93	332.1575	159.0364	5	5-100	0.998	12.8

446	Sethoxydim	$C_{17}H_{29}NO_3S$	$[M+H]^+$	17.21	328.1941	107.0491	10	10-200	0.991	8.9
447	Siduron	$C_{14}H_{20}N_2O$	$[M+H]^+$	8.68	233.1648	55.0542	5	5-100	0.9871	13.4
448	Silthiofam	$C_{13}H_{21}NOSSi$	$[M+H]^+$	13.43	268.1186	73.0468	5	5-100	0.9983	27.4
449	Simazine	$C_7H_{12}ClN_5$	$[M+H]^+$	5.04	202.0854	132.0323	5	5-100	0.998	11.4
450	Simetryn	$C_8H_{15}N_5S$	$[M+H]^+$	5.18	214.1121	68.0243	5	5-100	0.995	26.0
451	Sulfallate	$C_8H_{14}ClNS_2$	$[M+H]^+$	14.69	224.0329	88.0216	50	50-1000	0.9897	18.0
452	Sulfentrazone	$C_{11}H_{10}Cl_2F_2N_4O_3S$	$[M+H]^+$	6.43	386.9892	306.9944	5	5-100	0.9947	29.7
453	Sulfotep	$C_8H_{20}O_5P_2S_2$	$[M+H]^+$	15.8	323.03	96.9508	5	5-100	0.9939	17.5
454	Tebufenpyrad	$C_{18}H_{24}ClN_3O$	$[M+H]^+$	16.69	334.1681	117.0209	5	5-100	0.9987	21.8
455	Tebutam	$C_{15}H_{23}NO$	$[M+H]^+$	12.43	234.1852	91.0542	5	5-100	0.9978	12.1
456	Tebuthiuron	$C_9H_{16}N_4OS$	$[M+H]^+$	4.59	229.1118	172.0903	5	5-100	0.999	27.2
457	Temephos	$C_{16}H_{20}O_6P_2S_3$	$[M+H]^+$	17.8	466.997	124.9821	5	5-100	0.9892	9.5
458	Terbucarb	$C_{17}H_{27}NO_2$	$[M+H]^+$	15.81	278.2116	109.0648	5	5-100	0.9932	23.0
459	Terbuthylazine	$C_9H_{16}ClN_5$	$[M+H]^+$	8.9	230.1167	174.0541	5	5-100	0.9992	21.8
460	Tetraconazole	$C_{13}H_{11}Cl_2F_4N_3O$	$[M+H]^+$	11.92	372.029	70.04	5	5-100	0.9985	22.3
461	Tetramethrin	$C_{19}H_{25}NO_4$	$[M+H]^+$	17.09	332.1856	164.0706	5	5-100	0.9956	15.2
462	Thenylchlor	$C_{16}H_{18}ClNO_2S$	$[M+H]^+$	13.03	324.0819	127.0212	5	5-100	0.9963	24.1
463	Thiazafluron	$C_6H_7F_3N_4OS$	$[M+H]^+$	5.14	241.0365	74.0059	5	5-100	0.9987	29.7
464	Thiazopyr	$C_{16}H_{17}F_5N_2O_2S$	$[M+H]^+$	15.48	397.0997	377.0942	5	5-100	0.9902	19.3
465	Thiobencarb	$C_{12}H_{16}ClNOS$	$[M+H]^+$	15.23	258.0714	125.0153	5	5-100	0.9912	10.7
466	Thionazin	$C_8H_{13}N_2O_3PS$	$[M+H]^+$	8.14	249.0456	174.9726	5	5-100	0.9948	14.9
467	Tiocarbazil	$C_{16}H_{25}NOS$	$[M+H]^+$	18.31	280.173	91.0542	5	5-100	0.9964	24.7
468	Tolclofos-Methyl	$C_9H_{11}Cl_2O_3PS$	$[M+H]^+$	15.69	300.9616	124.9821	50	50-1000	0.9958	15.9
469	Triallate	$C_{10}H_{16}Cl_3NOS$	$[M+H]^+$	18.09	304.0091	142.9217	25	25-500	0.9949	23.7
470	Trichlorfon	$C_4H_8Cl_3O_4P$	$[M+H]^+$	3.36	256.9299	78.9945	25	25-500	0.9966	13.9
471	Triclopyricarb	$C_{15}H_{13}Cl_3N_2O_4$	$[M+H]^+$	16.68	391.0005	163.0628	10	10-200	0.9971	21.1
472	Trietazine	$C_9H_{16}ClN_5$	$[M+H]^+$	11.43	230.1167	71.0604	5	5-100	0.9974	10.0
473	Triflumizole	$C_{15}H_{15}ClF_3N_3O$	$[M+H]^+$	15	346.0929	69.0447	5	5-100	0.995	26.1
474	Triflumuron	$C_{15}H_{10}ClF_3N_2O_3$	$[M+H]^+$	14.57	359.0405	138.9938	10	10-200	0.9969	19.1
475	Tri-Isobutyl Phosphate	$C_{12}H_{27}O_4P$	$[M+H]^+$	14.54	267.173	98.9842	5	5-100	0.9939	26.9
476	Trinexapac-Ethyl	$C_{13}H_{16}O_5$	$[M+H]^+$	7.68	253.1071	69.0335	10	10-200	0.9995	10.8
477	Triphenyl phosphate	$C_{18}H_{15}O_4P$	$[M+H]^+$	15	327.0781	77.0386	5	5-100	0.9911	17.7
478	Uniconazole	$C_{15}H_{18}ClN_3O$	$[M+H]^+$	10.67	292.1213	70.04	5	5-100	0.9958	19.3
479	Vernolate	$C_{10}H_{21}NOS$	$[M+H]^+$	15.37	204.1414	86.06	25	25-500	0.9928	14.2
